# Adjuvant selection and optimization in the design of an epstein–barr virus–based multi-epitope vaccine against Burkitt lymphoma

**DOI:** 10.1186/s43046-026-00423-1

**Published:** 2026-10-27

**Authors:** Jesika Trimurti, Sari Eka Pratiwi, Syarifah Nurul Yanti Rizki Syahab Asseggaf, Ysrafil Ysrafil

**Affiliations:** 1 Universitas Tanjungpura, Pontianak, Indonesia; 2 Universitas Palangka Raya, Palangka Raya, Indonesia

**Keywords:** Immunology, Vaccines, Adjuvant, Immunoinformatics, Epstein-Barr virus, Burkitt lymphoma

## Abstract

**Background:**

This study aimed to identify and optimize a multi-epitope vaccine construct against Epstein–Barr virus (EBV)-associated Burkitt lymphoma, with particular emphasis on adjuvant selection, using an immunoinformatics approach.

**Methods:**

CTL, HTL, and B-cell epitopes derived from gp350, EBNA1, LMP2A, and BZLF1 were screened based on their predicted immunological properties. We assembled candidate epitopes into multi-epitope vaccine constructs with different configurations and evaluated them using immunoinformatics analyses.

**Results:**

A total of 39 CTL, 21 HTL, and 10 B-cell epitopes met the predefined selection criteria. Five multi-epitope vaccine constructs (R1–R5) were generated and compared. The 50 S ribosomal protein L7/L12 was selected as the adjuvant, and R5 was selected as the final construct based on its comparatively favorable predicted tertiary structure and molecular interactions with TLR2 and TLR4. R5 consisted of 790 amino acids and showed favorable predicted antigenicity (0.6479), solubility, non-allergenicity, and non-toxicity. The predicted population coverage was 98.46% worldwide. Molecular docking demonstrated favorable interactions with TLR2 and TLR4, while iMODS analysis indicated structural stability and favorable dynamic properties.Immune simulation predicted both cellular and humoral immune responses following three administrations at 28-day intervals.

**Conclusion:**

The L7/L12-adjuvanted R5 construct demonstrated favorable predicted immunological, structural, and molecular characteristics in silico. These findings support its consideration as a candidate for further experimental investigation against EBV-associated Burkitt lymphoma. However, experimental validation is required to determine its actual immunogenicity, safety, and protective efficacy.

**Supplementary Information:**

The online version contains supplementary material available at https://doi.org/10.1186/s43046-026-00423-1.

## Background

Burkitt lymphoma (BL) is a subtype of Non-Hodgkin lymphoma (NHL) that develops aggressively in germinal center B cells. The disease was first described as a jaw sarcoma in children in Uganda by Denis Burkitt in 1958. It primarily occurs in children in Central Africa and is often associated with Epstein-Barr Virus (EBV). Burkitt lymphoma was classified into three subtypes by the World Health Organization. The three subtypes are sporadic BL (sBL), endemic BL (eBL), and immunodeficiency-associated BL (ID-BL). The highest number of EBV-associated cases is detected in the eBL subtype, accounting for 95% [1].

EBV has a structure similar to that of other human herpesviruses (HHVs), known as the first tumorigenic virus in humans to be identified due to its ability to cause tumors. This virus is transmitted through saliva from one individual to another. To date, EBV vaccine development has advanced to phase I and II clinical trials, with most candidates designed as prophylactic vaccines targeting the major envelope glycoprotein gp350. Targeting gp350 is effective for preventing primary EBV infection by inducing neutralizing antibodies that block viral entry into B cells; however, this strategy offers limited benefit once latent infection is established, particularly in EBV-associated malignancies such as Burkitt lymphoma (BL) [2]. These limitations highlight the need for multi-epitope vaccine approaches that integrate both prophylactic and therapeutic components by combining epitopes derived from viral proteins expressed at different stages of the EBV life cycle.

Burkitt lymphoma is characterized by EBV latency I, a highly restricted latency program in which EBNA1 is the only viral protein consistently expressed in tumor cells [2]. EBNA1 plays an essential role in viral episome maintenance but also evades CD8⁺ T-cell recognition through its glycine–alanine repeat (GAr) domain, which limits proteasomal processing and MHC class I presentation [3]. Despite this immune evasion mechanism, EBNA1-derived epitopes are efficiently presented via MHC class II pathways and can elicit robust CD4⁺ T-cell responses, supporting its relevance as a therapeutic vaccine target [4].

LMP2A was included as a potential therapeutic target because, although it is not a characteristic component of the classical latency I program, its expression has been reported in a subset of EBV-positive BL cases [5]. LMP2A has been implicated in B-cell survival and signaling pathways relevant to EBV-associated tumor biology, including activation of the Ras/PI3K/AKT pathway.⁵ Thus, the inclusion of LMP2A was intended to broaden immune recognition toward BL cells exhibiting non-canonical EBV latency patterns rather than to represent the predominant viral expression profile of BL. LMP2A can enhance MYC-driven cell-cycle progression by promoting instability of the tumor suppressor protein p27Kip1. Furthermore, LMP2A signaling induces the Ras/PI3K/AKT pathway, which plays a crucial role in B-cell survival. LMP2A may also suppress MYC-induced apoptosis, partly through upregulation of the anti-apoptotic protein Bcl-XL, thereby allowing deregulated MYC-expressing B cells to survive and proliferate. Collectively, these mechanisms suggest that LMP2A may contribute to EBV-associated Burkitt lymphoma by promoting B-cell survival and supporting malignant progression [5]. Although BZLF1 is a lytic immediate-early protein and classical BL exhibits minimal lytic gene expression, sporadic lytic reactivation and abortive lytic cycles have been reported in EBV-associated tumors, potentially exposing lytic antigens to immune surveillance [1]. BZLF1 was included as an additional potential therapeutic antigen because it is an immediate-early EBV protein that functions as a key molecular switch initiating the transition from latency to the lytic cycle.⁶ Although classical BL is predominantly associated with latency I, BZLF1 expression and evidence of lytic-cycle activation have been detected in subsets of EBV-positive BL tumors, indicating heterogeneity in EBV gene-expression programs.⁶˒⁷ Therefore, BZLF1 may provide an additional antigenic target for immune recognition of EBV-infected tumor cells undergoing lytic reactivation [6]. Taken together, the inclusion of gp350 as a prophylactic antigen, together with EBNA1, LMP2A, and BZLF1 as potential therapeutic targets, was intended to broaden the immunological coverage of the vaccine across different stages and patterns of EBV infection, including viral entry, latent viral persistence, and episodic lytic reactivation. This strategy provides a rationale for integrating prophylactic and therapeutic components within a single multi-epitope vaccine design.

The multi-epitope vaccine construction strategy aims to induce a stronger immune response by activating cytotoxic T cells (CTL), T helper cells (HTL), and B-cells, thereby potentially reducing the risk of developing BL significantly. The development of a multi-epitope vaccine with prophylactic and therapeutic effects on cancer through immunoinformatics analysis has been successfully demonstrated in previous studies [7]. For a construction of a multi-epitope vaccine, not only is the selection of optimal epitopes necessary, but to increase the immunogenicity of the vaccine, the addition of adjuvants also needs to be considered. Adjuvants are important in increasing antigenicity, immunogenicity, stability, and long-lasting potential [8]. Therefore, this study developed the construction of an EBV vaccine with several adjuvants, followed by further analysis to obtain the best EBV multi-epitope vaccine candidate.

## Methods

**Fig. 1:**
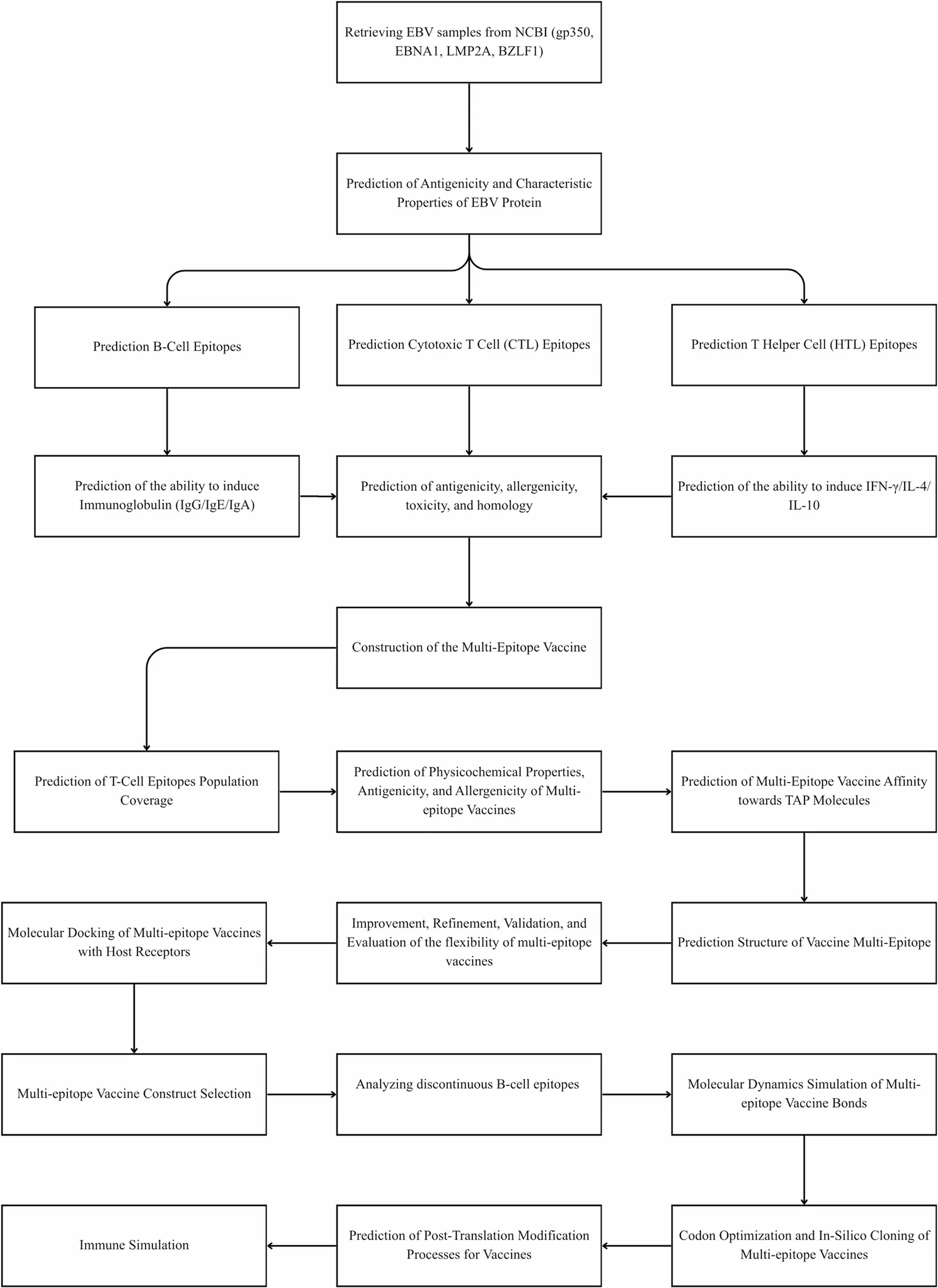
Workflow of the immunoinformatics approach used for the design and evaluation of the multi-epitope vaccine targeting Epstein–Barr Virus (EBV)-associated Burkitt lymphoma.EBV, Epstein-Barr Virus; CTL, cytotoxic T cell; HTL, helper T cell; IFN, interferon; IL, interleukin

The immunoinformatics approach used to design and evaluate the novel chimeric vaccine is detailed below, along with the online tools and databases utilized in this study (Fig. 1) (Table S1). 

### Retrieving EBV samples from NCBI

Samples of EBV proteins gp350 with accession (P03200), EBNA1 with accession (P03211), LMP2A with accession (YP_401631.1), and BZLF1 with accession (QAC12516.1) were obtained in the form of amino acid sequences accessible through the NCBI virus website (https://www.ncbi.nlm.nih.gov/).

### Prediction of antigenicity and characteristic properties of EBV protein

Prediction of the antigenicity of EBV protein samples using the Vaxijen v2.0 website (https://ddgpharmfac.net/vaxijen/VaxiJen/VaxiJen.html). Proteins were antigenic if the predicted result exceeded the specified threshold of 0.4. This threshold is in line with the VaxiJen validation model for viruses, where a value above 0.4 indicates an accuracy rate of 70% [9]. After that, the antigenic proteins were predicted using the ProtParam website (https://web.expasy.org/protparam/). This analysis focused on identifying proteins with optimal physicochemical properties, specifically those with a molecular weight of 40–110 kilodaltons (kDa), aliphatic index (66,5–84,3), instability index (< 40), and GRAVY index (< 0) [10]. These parameters were used to evaluate the structural stability and overall suitability of the proteins: molecular weight influences expression feasibility; the aliphatic index reflects thermostability; the instability index predicts in vitro stability; and the GRAVY value indicates hydrophilicity, which is associated with better solubility.

### Prediction of cytotoxic T cell (CTL) epitopes

The NetMHCpan EL 4.0 was used with the Immune Epitope Database (IEDB) http://tools.iedb.org/mhci/, to predict CTL epitopes. CTL epitopes are formulated to trigger cellular immune responses by interacting with MHC I. A predetermined epitope with a length of 9 amino acids is used to predict CTL epitopes. This length is the most commonly recognized amino acid length by MHC-I. This length is the most commonly recognized amino acid length by MHC-I. Furthermore, epitopes with percentile values ≤ 2 are analyzed for immunogenicity using IEDB, with results yielding a positive value considered immunogenic (≥ 0).

### Prediction of T helper cell (HTL) epitopes

HTL epitopes are predicted using the IEDB website (http://tools.iedb.org/mhci/). HTL epitopes are predicted using a predefined epitope length of 15 amino acids. This epitope length corresponds to the typical amino acid sequence recognized by MHC-II. HTL epitopes are designed to enhance humoral immune responses through their presentation by MHC II to HTL. HTL epitopes with a percentile value ≤ 2 were selected for further evaluation. Additional analyses were performed using the IFNepitope, IL4Pred, and IL10Pred servers to assess their potential to induce cytokine responses, specifically interferon (IFN)-γ, interleukin (IL)-4, and IL-10 production. These predictions were conducted to determine the immunomodulatory capacity of the selected HTL epitopes and to identify candidates capable of eliciting balanced cellular and humoral immune responses.

### Prediction B-cell epitopes

B-cell epitope prediction can be performed using the ABCpred website (http://crdd.osdd.net/raghava/abcpred/). A predetermined epitope of 16 amino acids is used to predict B-cell epitopes using the Chou and Fasman beta-turn prediction method. This length is based on results from several tests, which showed the best performance with an accuracy of 66%, making it optimal for predicting B-cell epitopes compared to other lengths [7]. Epitopes with values ≥ 0.51 were collected and proceeded for antigenicity, allergenicity, toxicity, homology, and immunoglobulin-class prediction.

### Prediction of CTL, HTL, and B-cell epitope properties

All of CTL, HTL, and B-Cell epitopes were predicted for antigenicity, allergenicity, toxicity, and homology. The antigenicity of epitopes is predicted using the VaxiJen v2.0 website (http://www.ddg-pharmfac.net/vaxijen/VaxiJen/VaxiJen.html), and a result of ≥ 0.4 indicates that the epitope is antigenic [9]. Next, the allergenicity properties of vaccine epitopes are predicted using the Algpred 2.0 server (https://webs.iiitd.edu.in/raghava/algpred2/algo.html). Allergenicity predictions are acceptable if both websites indicate that the vaccine epitope is non-allergenic; the toxicity properties of vaccine epitopes are predicted using the ToxinPred website (https://webs.iiitd.edu.in/raghava/toxinpred/protein.php). For homology prediction using NCBI BLAST (https://blast.ncbi.nlm.nih.gov/Blast.cgi), the search was restricted to *Homo sapiens* (taxid: 9606) to assess potential sequence similarity between the selected epitopes and human proteins. The analysis was based on the BLAST E-value, with a threshold of > 0.05. Epitopes with an E-value > 0.05 were considered to show no significant sequence similarity to human proteins and were retained for subsequent vaccine construction.

For B-cell epitopes, immunoglobulin-class prediction was additionally performed using the IgPred web server (https://webs.iiitd.edu.in/raghava/igpred/). The predicted immunoglobulin classes were interpreted as computational predictions of association with IgA, IgE, or IgG responses and were not considered direct evidence of antibody induction. Throughout the selection process, only CTL, HTL, and B-cell epitopes that demonstrated antigenic properties, while being predicted as non-allergenic, non-toxic, and non-homologous, were considered eligible for incorporation into the vaccine construct.

### Construction of the multi-epitope vaccine

Twenty constructs were created and grouped into five types, differentiated by three adjuvants and one without an adjuvant. Each construct has a different number of epitopes, with epitope selection based on percentile values. Percentile values are the primary focus in epitope selection due to their ability to bind to MHC complexes. For CTL and HTL epitopes, those with the smallest percentile score were prioritized. For LBL epitopes, those with the largest percentile were selected. These constructs were subsequently assessed for their antigenicity, allergenicity, toxicity, and physicochemical properties. The CTL, HTL, and B-cell epitopes were constructed by starting with an adjuvant, then linking using EAAAK linkers, GPGPG (Gly-Pro-Gly-Pro-Gly), AAY (Ala-Ala-Tyr), KK (Lys-Lys), and ending with a 6x His-tag [7, 11, 12]. In multi-epitope vaccine design, the incorporation of appropriate linkers is equally important, as they help preserve the structural and functional integrity of individual epitopes while preventing the formation of junctional epitopes that may compromise immunogenic specificity. These linkers also enable the covalent attachment of adjuvants to selected epitopes, facilitating the construction of a structurally integrated epitope-based vaccine. Given that epitope-based vaccines generally exhibit lower intrinsic antigenicity compared to live attenuated vaccines, the strategic inclusion of adjuvants and linkers is essential to enhance their overall immunogenic potential [13].

In this construct, different linkers were selected based on their established immunological roles. The AAY linker was employed to connect CTL epitopes, as it has been reported to facilitate proteasomal processing and improve epitope presentation, thereby enhancing immunogenicity and peptide stability. The GPGPG linker was used between HTL epitopes; previous studies, including those by Livingston et al., have demonstrated its ability to promote effective T helper cell responses and support MHC-II presentation. For B-cell epitopes, KK linkers were incorporated to maintain appropriate epitope separation and contribute to improved immune recognition. Additionally, the rigid EAAAK linker was applied to link the adjuvant with CTL epitopes, as it maintains a stable spatial distance between functional domains. This rational arrangement ensures that both the adjuvant and epitopes retain their respective functional properties within the final vaccine construct [14–16].

**Fig. 2:**
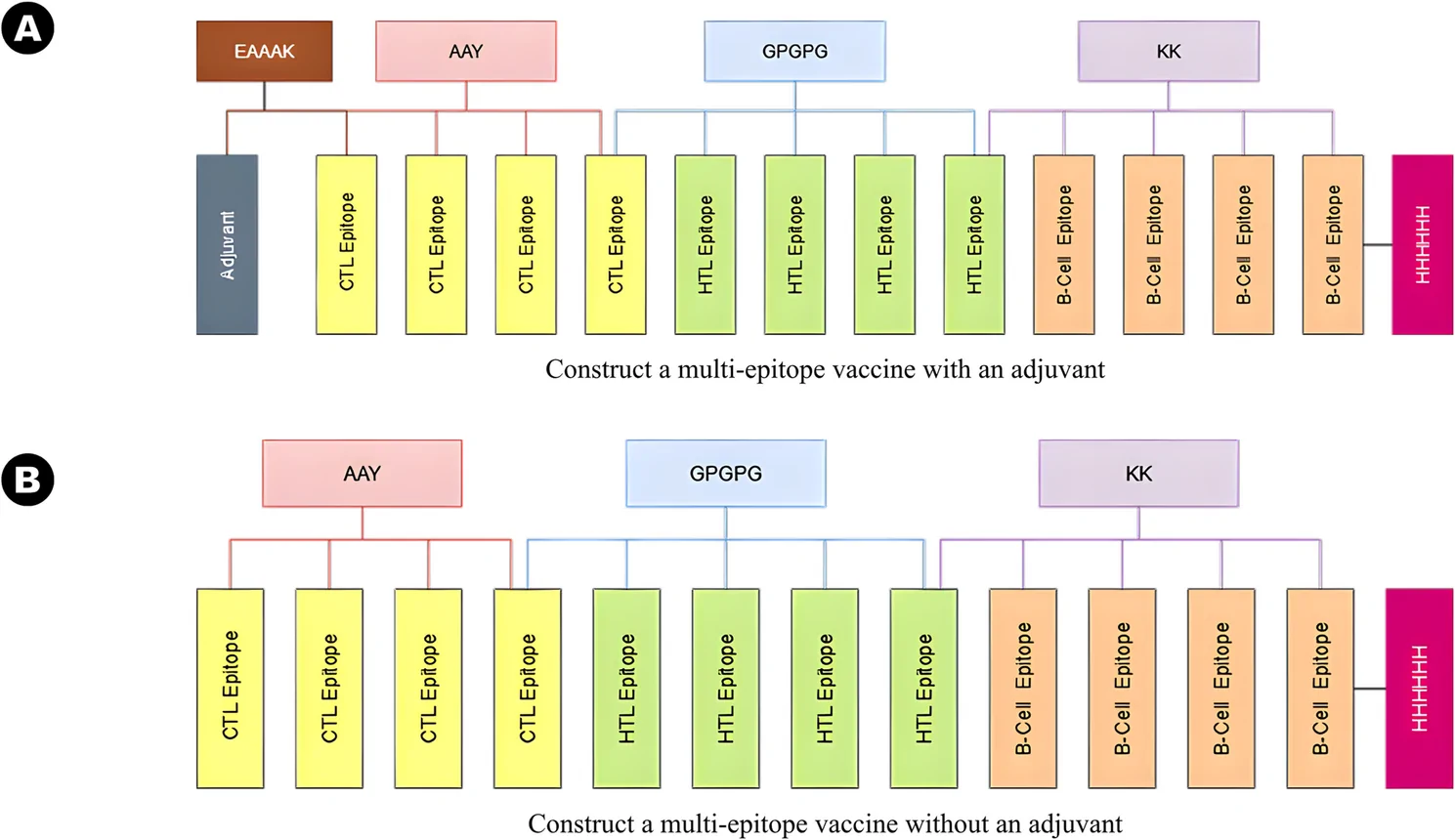
Schematic representation of the designed multi-epitope vaccine constructs. A Multi-epitope vaccine construct containing an adjuvant; B Multi-epitope vaccine construct without an adjuvant. The diagram illustrates the structural organization of selected CTL (yellow), HTL (green), and B-cell (orange) epitopes linked using specific linkers: AAY (red) for CTL epitopes, GPGPG (blue) for HTL epitopes, and KK (purple) for B-cell epitopes. The adjuvant sequence is shown in grey and the His-tag in pink. HTL, helper T lymphocyte; CTL, cytotoxic T lymphocyte

The adjuvant used in this study is OV ASP-1 (Onchocerca volvulus activation-associated secreted protein-1) with accession code (O16854), 50 S ribosomal protein L7/L12 (Locus RL7_MYCTU) with accession code (P9WHE3.1), and human beta-defensin-3 with accession code (Q5U7J2). (Fig. 2). 

### Prediction of T-cell epitopes population coverage

Predictions of T-Cell epitope coverage worldwide were obtained from the IEDB Population Coverage website (http://tools.iedb.org/population/). The analysis included multiple geographic regions, namely Australia, Europe, North Africa, Sub-Saharan Africa, North America, Central and South America, Oceania, Northeast Asia, Southeast Asia, South Asia, and West Asia. This evaluation was conducted to assess the worldwide distribution of Human Leukocyte Antigen (HLA) alleles associated with MHC-I and MHC-II molecules and to estimate the proportion of individuals in different populations who may respond to the selected epitopes. Ultimately, this approach supports the assessment of the vaccine candidate’s potential prophylactic and therapeutic applicability across diverse global populations.

### Prediction of physicochemical properties, antigenicity, and allergenicity of multi-epitope vaccines

The prediction of multi-epitope vaccine properties was performed sequentially based on physicochemical properties, antigenicity, and allergenicity. Physicochemical properties were determined using the ProtParam website. The antigenicity of each epitope is predicted using the VaxiJen v.2.0 website. Next, the allergenicity of the multi-epitope vaccine epitope is predicted using the Algpred 2.0 website. Finally, solubility is predicted using SOLpro (http://scratch.proteomics.ics.uci.edu), which predicts solubility based on a probability of ≥ 0.5.

### Analysis of the TAP molecules binding capacity

Affinity analysis of construct vaccines toward TAP (Transporter Associated with Antigen Processing) molecules using the TAPPred website (https://webs.iiitd.edu.in/raghava/tappred/). The complete amino acid sequence of each vaccine construct, rather than individual CTL epitopes, was submitted for TAP-binding analysis to assess the predicted TAP-binding characteristics of the constructs [7]. This analysis was performed as a downstream characterization of the vaccine constructs and was not used as an independent criterion for CTL epitope selection.

### Prediction structure of vaccine multi-epitope

The prediction of multi-epitope vaccine structures aims to determine the secondary and tertiary structures of multi-epitope vaccines to analyze the molecular dynamics of vaccine binding to Toll-Like receptor (TLR). Secondary structure prediction of multi-epitope vaccines using the PSIPRED (http://bioinf.cs.ucl.ac.uk/psipred/ ) and SOPMA https://npsa-prabi.ibcp.fr/cgi-bin/npsa_automat.pl?page=/NPSA/npsa_sopma.html). Next, the tertiary structure of the multi-epitope vaccine is predicted using the trRosetta website (https://yanglab.nankai.edu.cn/trRosetta/). Then followed by visualization of the 3D structure of the vaccine using PyMOL application.

### Improvement, refinement, validation, and evaluation of the flexibility of multi-epitope vaccines

The tertiary structure of the construct vaccine was refined using the GalaxyRefine website. The refined vaccine structure model selected was model 1 of the 5 models generated because it had the highest quality. Next, the selected multi-epitope vaccine protein structure was analyzed using ProSA (https://prosa.services.came.sbg.ac.at/prosa.php), ERRAT (https://saves.mbi.ucla.edu/), and PROCHCEK (https://saves.mbi.ucla.edu/). Finally, the flexibility of the vaccine was evaluated using the CABSFLEX 2.0 website (http://biocomp.chem.uw.edu.pl/CABSflex2/submit).

### Molecular docking of multiepitope vaccines with TLRs 2 and 4

The refined 3D vaccine structure was docked with APC receptors to validate its potential to induce an immune response via the TLR pathway. In this study, TLR2 (PDB ID: 2Z7X) and TLR4 (PDB ID: 4G8A) data were obtained from the RCSB PDB website (The receptor structures were used as obtained from the PDB, and no manual chain selection or modification of the receptor structures was performed prior to docking(https://cluspro.bu.edu/home.php) and HDOCK (http://hdock.phys.hust.edu.cn/) with their default settings. For ClusPro 2.0, the generated docking models were evaluated based on the number of cluster members, representative models, and weighted scores. The representative model with the lowest weighted score among the selected docking clusters was used for subsequent structural and interaction analyses. For HDOCK, the generated models were evaluated according to their docking scores and confidence scores. A confidence score ≥ 0.7 was considered a favorable computational prediction of the docking model and was not interpreted as direct evidence of high binding affinity. The selected docking complexes were subsequently analyzed to characterize the predicted vaccine-TLR interaction interfaces, including interacting residues and non-covalent interaction types. These interactions were used to describe the structural compatibility of the vaccine construct with the receptor models.

TLR2 and TLR4 were selected as docking targets based on their roles as pattern-recognition receptors involved in innate immune signaling. TLR2 has been implicated in the recognition of various microbial and viral components, including viral glycoproteins, whereas TLR4 is involved in innate immune signaling and downstream inflammatory responses. The selection of TLR4 was also related to the inclusion of 50 S ribosomal protein L7/L12 as the adjuvant in the vaccine construct, which was selected based on its reported TLR4 agonistic activity. Therefore, docking of the complete multi-epitope vaccine construct with TLR2 and TLR4 was performed to computationally assess its predicted structural interactions with these innate immune receptors. Because the docking analysis used the complete vaccine construct containing the L7/L12 adjuvant, EBV-derived epitopes, and linkers, the docking results were interpreted as interactions of the assembled construct rather than as evidence of binding or activation attributable to any individual component. The docking analysis was not considered direct evidence of TLR2/TLR4 activation or subsequent adaptive immune responses [13].

### Multi-epitope vaccine construct selection

The selection of the multi-epitope vaccine construct was carried out through a comprehensive evaluation process. Several parameters were taken into consideration, including antigenicity to ensure the ability to elicit an immune response, allergenicity and toxicity to confirm safety, as well as physicochemical characteristics to assess structural stability and suitability for expression. In addition, the predicted tertiary structure of the protein was analyzed to evaluate conformational integrity, and molecular docking was performed to determine its binding affinity toward Toll-like receptors TLR2 and TLR4. Collectively, these criteria were applied to identify a vaccine construct with optimal immunogenic potential, structural stability, and safety profile.

### Analyzing discontinuous B-cell epitopes

Analyzing discontinuous B-cell epitopes within the three-dimensional (3D) structure of a vaccine is important to understand antigen–antibody interactions better. In this study, epitope identification was performed using the ElliPro web server (https://tools.iedb.org/ellipro/), which predicts and visualizes antibody epitopes based on the 3D structure using Thornton’s method. The predicted epitopes were subsequently visualized with the Jmol viewer. Default parameters were applied, with a minimum score of 0.5 and a maximum distance of 6 Å [7].

### Normal mode analysis of the multi-epitope vaccine construct

The molecular docking results were further analyzed using the iMODS server (http://imods.chaconlab.org) to evaluate the structural dynamics of the multi-epitope vaccine. Normal mode analysis (NMA) was performed to assess the deformability and flexibility of the vaccine structure. The resulting deformability profile was used to identify regions of the vaccine structure with greater or lower flexibility, while the eigenvalue provided an indication of the energy required for structural deformation.

### Codon optimization and in-silico cloning of multi-epitope vaccines

Codon optimization was performed using the IDT Codon Optimization Tool (https://www.idtdna.com/pages/tools/codon-optimizationtool), with *Pichia pastoris* selected as the expression host. *P. pastoris* was selected because of its established use for heterologous recombinant protein production. The nucleotide sequence of the multi-epitope vaccine construct was adapted according to the codon usage preferences of *P. pastoris*. The optimized nucleotide sequence was subsequently used for in silico cloning using SnapGene v4.2 (https://www.snapgene.com/), with pPICZαA used as the expression vector. The vaccine coding sequence was inserted between the BsaAI and NotI restriction sites to generate the recombinant plasmid.

### Prediction of post-translation modification processes for vaccines

Prediction of post-translational modifications (PTM) of vaccines using the MusiteDeep website (https://www.musite.net/). In this study, the modification processes evaluated were phosphorylation (P), glycosylation (gl), ubiquitination (ub), acetylation (ac), methylation (me), and hydroxylation (hy).

### Immune simulation

The immune response to the multi-epitope vaccine construct was simulated using the C-IMMSIM server (https://kraken.iac.rm.cnr.it/C-IMMSIM/index.php?page=1), to evaluate its predicted cellular and humoral immunogenicity following different multi-dose immunization strategies. hree sequential injections were administered at 28-day intervals. The simulation parameters were set as follows: simulation volume = 10 and simulation steps = 1,095, corresponding to a 365-day simulation period, while the random seed and host HLA selection were maintained at default settings. The injected antigen consisted of the vaccine construct without LPS, combined with an adjuvant dose of 100 units, and the number of antigen particles per injection was set to 1,000. The resulting immune responses were evaluated based on predicted antibody production, B-cell and T-cell responses, as well as cytokine and interleukin secretion [17].

## Results

### CTL epitope prediction

The CTL epitopes gp350, EBNA1, LMP2A, and BZLF1 of EBV, analyzed through IEDB, were selected based on percentile score ≤ 0.2, a length of 9 amino acids, and immunogenicity (≥ 0). Therefore, they exhibited good antigenicity (≥ 0.4), non-toxic, non-allergenic, and non-homologous, supporting their potential as promising candidates for multi-epitope vaccine construction. In total of 140, 57, 135, and 43 with percentile score ≤ 0.2, a length of 9 amino acids, and immunogenicity (≥ 0) were identified from gp350, EBNA1, LMP2A, and BZLF1, respectively. From the analysis, 8 epitopes from gp350, 2 epitopes from EBNA1, 27 epitopes from LMP2A, and 2 epitopes from BZLF1 were antigenic, non-toxic, non-allergenic, and non-homologous. (Table S2) (Table S3) (Table S4) (Table S5).

### HTL epitope prediction

The HTL epitopes gp350, EBNA1, LMP2A, and BZLF1 of EBV, analyzed through IEDB, were selected based on a percentile score ≤ 0.2, a length of 15 amino acids. Therefore, they exhibited good antigenicity (≥ 0.4), non-toxic, non-allergenic, non-homologous, and able to induce IFN- γ/IL-4/IL-10. A total of 122, 48, 32, and 62 with percentile score ≥ 0.2, a length of 15 amino acids, were identified from gp350, EBNA1, LMP2A, and BZLF1, respectively. From the analysis, 6 epitopes from gp350, 2 epitopes from EBNA1, 5 epitopes from LMP2A, and 8 epitopes from BZLF1 were antigenic, non-toxic, non-allergenic, non-homologous, and able to induce IFN- γ/IL-4/IL-10.(Table S6) (Table S7) (Table S8) (Table S9).

### B-cell epitope prediction

B-cell epitopes from EBV proteins gp350, EBNA1, LMP2A, and BZLF1 were predicted using the ABCpred server, with percentile score ≥ 0.51 and epitope lengths of 16 amino acid sequences. Furthermore, B-cell epitopes were predicted based on toxicity, allergenicity, antigenicity, immunoglobulin (IgG, IgA, and IgE), and homology. A total of 501, 447, 170, and 115 with percentile score ≥ 0.51, and a length of 16 amino acids, were identified from gp350, EBNA1, LMP2A, and BZLF1, respectively. From the analysis, no epitopes from gp350, 7 epitopes from EBNA1, 1 epitope from LMP2A, and 2 epitopes from BZLF1 were antigenic, non-toxic, non-allergenic, non-homologous, and able to induce IgA/IgE/IgG. (Table S10). Therefore, B-cell epitopes from the gp350 protein will not be used in the development of multi-epitope vaccine constructs.

### Construction of the multi-epitope vaccine

Twenty constructs were designed and classified into four groups, distinguished by the presence of three different adjuvants and one without an adjuvant, with five constructs in each group. The adjuvant used in this study is OV ASP-1, 50 S ribosomal protein L7/L12 (Locus RL7_MYCTU), and human beta-defensin-3. Construction vaccine also consists of linkers, CTL epitopes, HTL epitopes, LBL epitopes, and his-tags. Each construct has a different number of epitopes, with epitope selection based on percentile values. Percentile values are the primary focus in epitope selection due to their ability to bind to MHC complexes. For CTL and HTL epitopes, those with the smallest percentile score were prioritized. For LBL epitopes, those with the largest percentile were selected. (Table 1) (Table 2).

**Table 1:** Construction vaccine multi-epitope

Adjuvant	Sequence	Linkers				His-tag
		Adjuvant	CTL	HTL	B-cell	
50 S ribosomal L7/L12	MAKLSTDELLDAFKEMTLLELSDFVKKFEETFEVTAAAPVAVAAAGAAPAGAAVEAAEEQSEFDVILEAAGDKKIGVIKVVREIVSGLGLKEAKDLVDGAPKPLLEKVAKEAADEAKAKLEAAGATVTVK	EAAAK	AAY	GPGPG	KK	HHHHHH
OV-ASP-1	MILFIIFPAIVVAVTGYNCPGGKLTALERKKIVGQNNKYRSDLINGKLKNRNGTYMPRGKNMLELTWDCKLESSAQRWANQCIFGHSPRQQREGVGENVYAYWSSVSVEGLKKTAGTDAGKSWWSKLPKLYENNPSNNMTWKVAGQGVLHFTQMAWGKTYKIGCGVATQCDGGRTLIVICHYSPGGNMVGEVIYHRGNPCKVDKDCYTKKCLSKSGLCRK	EAAAK	AAY	GPGPG	KK	HHHHHH
Human beta-defensin-3	GIINTLQKYYCRVRGGRCAVLSCLPKEEQIGKCSTRGRKCCRRKK	EAAAK	AAY	GPGPG	KK	HHHHHH
-	-	-	AAY	GPGPG	KK	HHHHHH

**Table 2:** Model construct vaccine multi-epitope

Model	Adjuvan	CTL	HTL	B-cell
1	50 S ribosomal L7/L12	4 epitopes	4 epitopes	4 epitopes
	OV-ASP-1			
	Human beta-defensin-3			
	Without adjuvant			
2	50 S ribosomal L7/L12	8 epitopes	8 epitopes	5 epitopes
	OV-ASP-1			
	Human beta-defensin-3			
	Without adjuvant			
3	50 S ribosomal L7/L12	11 epitopes	11 epitopes	6 epitopes
	OV-ASP-1			
	Human beta-defensin-3			
	Without adjuvant			
4	50 S ribosomal L7/L12	12 epitopes	14 epitopes	7 epitopes
	OV-ASP-1			
	Human beta-defensin-3			
	Without adjuvant			
5	50 S ribosomal L7/L12	14 epitopes	17 epitopes	8 epitopes
	OV-ASP-1			
	Human beta-defensin-3			
	Without adjuvant			

### Prediction T cell epitope population coverage

Prediction of T cell epitopes (CTL and HTL) population coverage was performed using a combined MHC-I and MHC-II prediction method. The global population coverage of all CTL epitopes and HTL epitopes for Model 1 (78.79%), Model 2 (94.39%), Model 3 (97.11%), Model 4 (97.74%), and Model 5 (98.46%). A closer examination of the HLA distribution revealed that the overall coverage is not driven by a single allele, but rather by a combination of highly prevalent class I and class II alleles. Among class I molecules, HLA-A*02 variants (A*02 :01, A*02:03, A*02:06), HLA-A*30:01, HLA-A*68:01/68:02, HLA-B*53:01, HLA-B*58:01, and HLA-B*57:01 contributed substantially to the projected coverage. Several of these alleles, particularly HLA-B*53:01 and HLA-A*30:01, are known to be frequently observed in African populations. For class II, alleles such as HLA-DRB*115:01, HLA-DRB*107:01, HLA-DRB*111:01, and DPA1/DPB1 combinations further enhanced the predicted helper T-cell coverage. Together, this allele diversity underlies the broad population representation achieved by the proposed multi-epitope construct. (Table S11)

### Evaluation characteristic of construction vaccine multi-epitope

The constructs were predicted based on antigenicity, allergenicity, toxicity, physicochemical properties, solubility, and population coverage. After analysis, only the vaccine construct with the sTable 50 S ribosomal L7/L12 protein adjuvant was selected for further prediction. After analysis, only the vaccine construct with the sTable 50 S ribosomal L7/L12 protein adjuvant was selected for the next analysis. The selected constructs were R2, R3, R4, and R5. (Table 3) (Table S12) (Table S13) (Table S14).

**Table 3:** Properties of the multiepitope vaccine construction with the 50 S ribosomal L7/L12 adjuvant

Multi-epitope Vaccine Properties		Multi-epitope Vaccine Design				
		R1	R2	R3	R4	R5
Length		320	483	586	688	790
Antigenicity		0,5657 (Antigenic)	0,6318 (Antigenic)	0,6409 (Antigenic)	0,6446 (Antigenic)	06479 (Antigenic)
Allergenicity		Non-allergen	Non-allergen	Non-allergen	Non-allergen	Non-allergen
Toxicity		Non-toxic	Non-toxic	Non-toxic	Non-toxic	Non-toxic
Physical and Chemical properties	Molecular weight	33991.94	51164.13	62103.20	72496.98	82914.81
	Instability index	30,43 (Stable)	29,15 (Stable)	34,33 (Stable)	33,43 (Stable)	32.51 (Stable)
	Aliphatic index	79,09 (Thermostable)	71,61 (Thermostable)	67,87 (Thermostable)	69,30 (Thermostable)	68,63 (Thermostable)
	GRAVY	-0,413	-0,481	-0,544	-0,481	-0,47
Solubility		Soluble	Soluble	Soluble	Soluble	Soluble
Population coverage	World	78,79%	94,39%	97.11%	97.74%	98.46%
	Indonesia	50,44%	83,9%	89.11%	89.3%	89.79%

Green, Construct that was continued; Red, Multi-epitope vaccine construct that was not continued

### Analysis of the TAP molecules binding capacity

The affinity of the R2, R3, R4, and R5 multi-epitope vaccine constructs toward the TAP molecule was predicted using the Tappred website. In construct R2, which consists of 483 amino acids, 150 amino acids (31%) exhibited high affinity, and 219 amino acids (37.3%) showed intermediate affinity. Construct R3, containing 586 amino acids, had 164 amino acids (27.9%) with high affinity and 227 amino acids (38.7%) with intermediate affinity. In construct R4, which consists of 688 amino acids, 216 amino acids (31.39%) exhibited high affinity, and 294 amino acids (42.7%) showed intermediate affinity. Construct R5, containing 790 amino acids, had 246 amino acids (31.1%) with high affinity and 339 amino acids (42.9%) with intermediate affinity. These predictions were considered as an additional in silico characterization of the vaccine constructs and were not used as an independent criterion for selecting the final CTL epitopes or vaccine construct.

### Prediction of the secondary and tertiary structure of the multi-epitope vaccine

Prediction of secondary structure using the PSIPRED and SOPMA websites. Both platforms, which report prediction accuracies above 80%, were employed under default parameters to ensure reliable estimation of structural elements. Secondary structure analysis provides insight into the local folding patterns of the polypeptide chain and is essential for understanding protein stability and functional conformation. Moreover, this analysis serves as a foundational step before tertiary structure modeling, as it reflects the intrinsic arrangement and interaction of amino acid residues within the construct. The predicted structural components included α-helices, extended strands (β-sheets), β-turns, and random coil regions, thereby offering a comprehensive overview of the vaccine candidate’s structural organization [18, 19]. **(**Table 4)(Figure S1.)

**Table 4:** Secondary structure and tertiary structure of the Multi-epitope vaccine

Secondary structure		Design Vaksin Multi-epitope			
		R2	R3	R4	R5
PSIPRED	Alpha Helix	12,39%	34,1%	35%	33,92%
	Extended strand	37,8%	10,58%	9,4%	9,49%
	coil	49,79%	55,29	55,52%	59,49%
SOPMA	Alpha helix	35,74%	30,20%	20,64%	22,41%
	Extended strand	16,94%	18.26%	14,68%	14,18%
	Beta turn	4,55%	4,27%	3,05%	3,16%
	Random coil	42,77%	47,27%	61,63%	60,25%

Trrosetta					
Vaccine Multi-epitope teritary	Model	TM score			
R2	1	0,332			
R3	1	0,357			
R4	1	0,141			
R5	1	0,140			

For tertiary structure, use trRosetta, which generated five models, and selected the model with the highest TM value. A TM value > 0.5 usually indicates a model of high quality. Based on the structural prediction generated by trRosetta, the tertiary structures of the multi-epitope vaccine constructs indicate that none of the four constructs meet the criteria for high-quality models. Subsequently, the four constructs were visualized using PyMOL.

### Improvement, refinement, validation, and evaluation of the flexibility of multiepitope vaccines

**Fig. 3:**
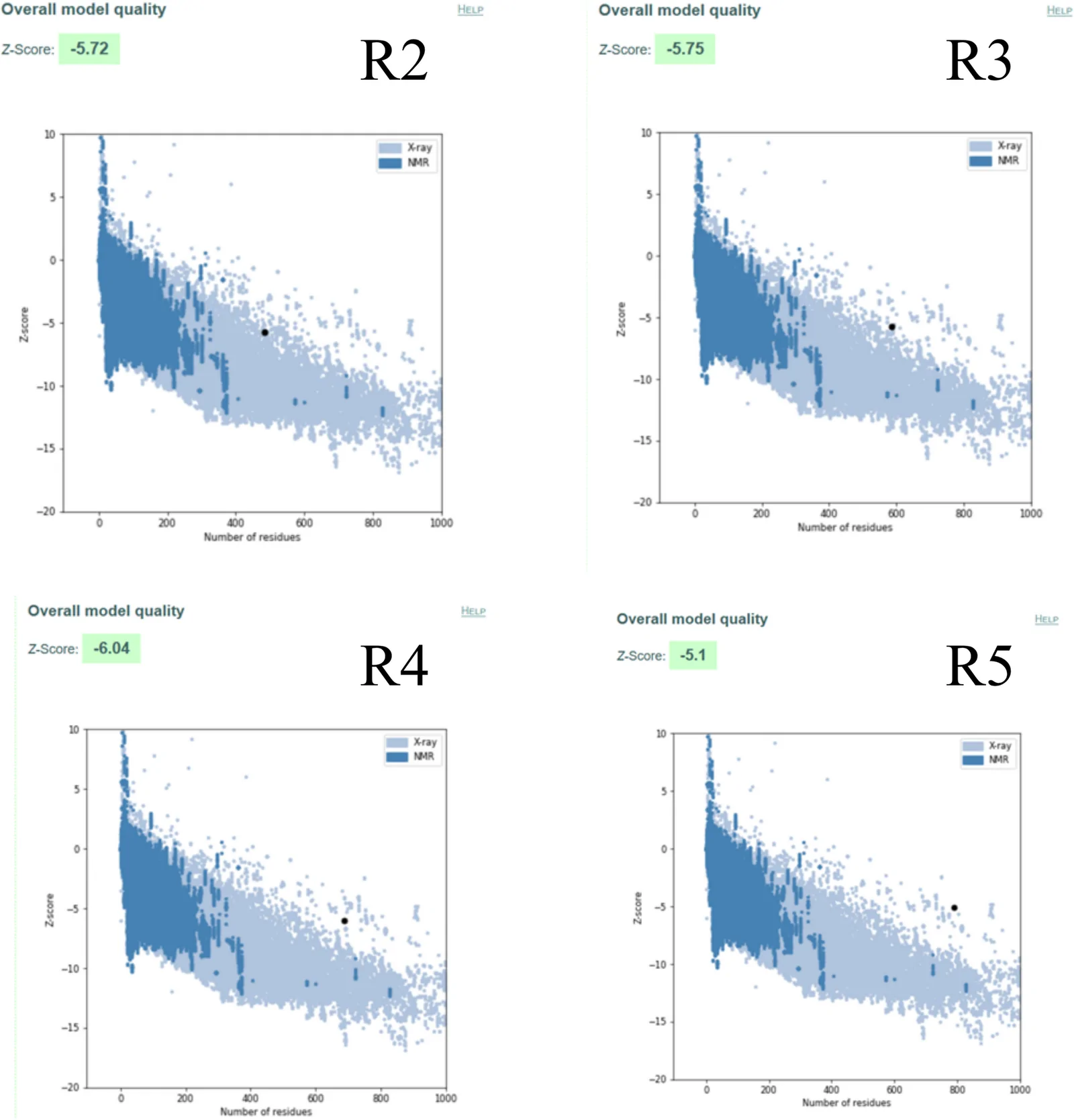
PROSA the Multi-Epitope Vaccine’s Tertiary Structure. ProSA-web validation of the refined models (R2–R5). The overall model quality is represented by the Z-scores obtained for each refined structure: R2 (− 5.72), R3 (− 5.75), R4 (− 6.04), and R5 (− 5.1). The Z-score values indicate the overall structural quality of the predicted 3D models compared to experimentally determined protein structures.

The GalaxyRefine website is used to refine the tertiary structure of the vaccine and produce the five best models. Then, from those five models, the best model is selected based on the criteria of GDT-HA, RMSD, MolProbity, Clash score, Poor rotamers, and Rama favored (Table 5**)**. ProSA, ERRAT, and PROCHECK were used to evaluate the comprehensive quality of all refined structural models (Table 6). The results obtained from ProSA showed Z-score values (Fig. 3**)**. The predictions generated by ERRAT displayed varying values. The Ramachandran plot generated by PROCHECK showed that several residues were located within the preferred region. In addition, the root mean square fluctuation (RMSF) value is predicted with CABSFLEX. (Figure S2.)(Table S15)(Table S16).

**Table 5:** Refining the Multi-epitope vaccine’s tertiary structure

Vaksin Multi-epitop	GDT-HA	RMSD	MolProbity	Clash score	Poor rotamers	Rama favored
R2 (Model 2)	0,9592	0,384	0,990	2,2	0,8	98,8
R3 (Model 3)	0,9697	0,358	1,103	3,1	0,2	99,0
R4 (Model 1)	0,9517	0,400	1,055	2,7	0	98,1
R5 (Model 2)	0,9589	0,391	1,013	2,3	0,2	99,1

**Table 6:** Validation of the Multi-epitope vaccine’s tertiary structures

Multi-epitope Vaccine	Model	PROSA	ERRAT	PROCHECK
R2	2	-5,72	98,4733	98,2%
R3	3	-5,75	95,2991	97,5%
R4	1	-6,04	96,3801	97,8%
R5	2	-5,1	98,0806	98,2%

### Molecular docking of multi-epitope vaccines with TLRs 2 and 4

The tertiary design of the improved vaccine was followed by affinity prediction between receptors (TLR2 and TLR4) using the ClusPro 2.0 and HDOCK server. The ClusPro prediction results for the R2 multi-epitope vaccine construct yielded 26 binding models with TLR2 and 29 binding models with TLR4. The R3 construct yielded 25 binding models with TLR2 and 28 binding models with TLR4. The R4 construct yielded 30 binding models with TLR2 and 30 binding models with TLR4. Finally, the R5 construct yielded 30 binding models with TLR2 and 24 binding models with TLR4. (Table S17) (Table S18) (Figure S3.) 

The predicted binding affinity between the designed vaccine construct and TLR2/TLR4 was evaluated using the HDOCK server. Docking scores were categorized as high (> 0.7), intermediate (0.5–0.7), and low (< 0.5) predicted binding affinity. The results illustrate the comparative interaction profiles of the vaccine construct with both receptors.

### Multi-epitope vaccine construction selection

Constructs were selected according to criteria consisting of antigenicity, allergenicity, toxicity, physicochemical properties, tertiary protein structure, and affinity with receptors TLRs 2 and 4. Based on prediction, all four vaccine constructs are antigenic, non-allergenic, non-toxic, and have favorable physicochemical properties. However, further prediction processes show that the R5 vaccine construct is better based on tertiary structure predictions and affinity to receptors TLRs 2 and 4. Therefore, the R5 vaccine construct was selected to proceed to molecular dynamics predictions. (Table 7**)**

**Table 7:** Comparison of multi-epitope vaccine constructs

Characterictic		Multi-epitope vaccine			
		R2	R3	R4	R5
Antigenicity		Antigenic	Antigenic	Antigenic	**Antigenic**
Allergenicity		Non-allergenic	Non-allergenic	Non-allergenic	**Non-allergenic**
Toxicity		Non-toxic	Non-toxic	Non-toxic	**Non-toxic**
TAP	High Affinity	(31%)	(27,9%)	(31,39%)	**(31**,**13%)**
	Intermediate Affinity	(37,3%)	(38,7%)	(42,7%)	**(42**,**9%)**
Physicochemistry		*Favourable*	*Favourable*	*Favourable*	***Favourable***
Tertiaritary Structure	ERRAT	98,4733	95,2991	96,3801	**98**,**0806**
	PROCHECK	98,2%	97,5%	97,8%	**98**,**2%**
	Cabsflex	3,359944	3,653882	2,367962	**3**,**151143038**
Affinity	TLR2	0,9702	0,9893	0,9963	**0**,**9819**
	TLR4	0,9552	0,9783	0,9565	**0**,**9931**

In bold, multi-epitope vaccine construct to be continued in subsequent predictions

### Analyzing discontinuous B-cell epitopes

**Fig. 4:**
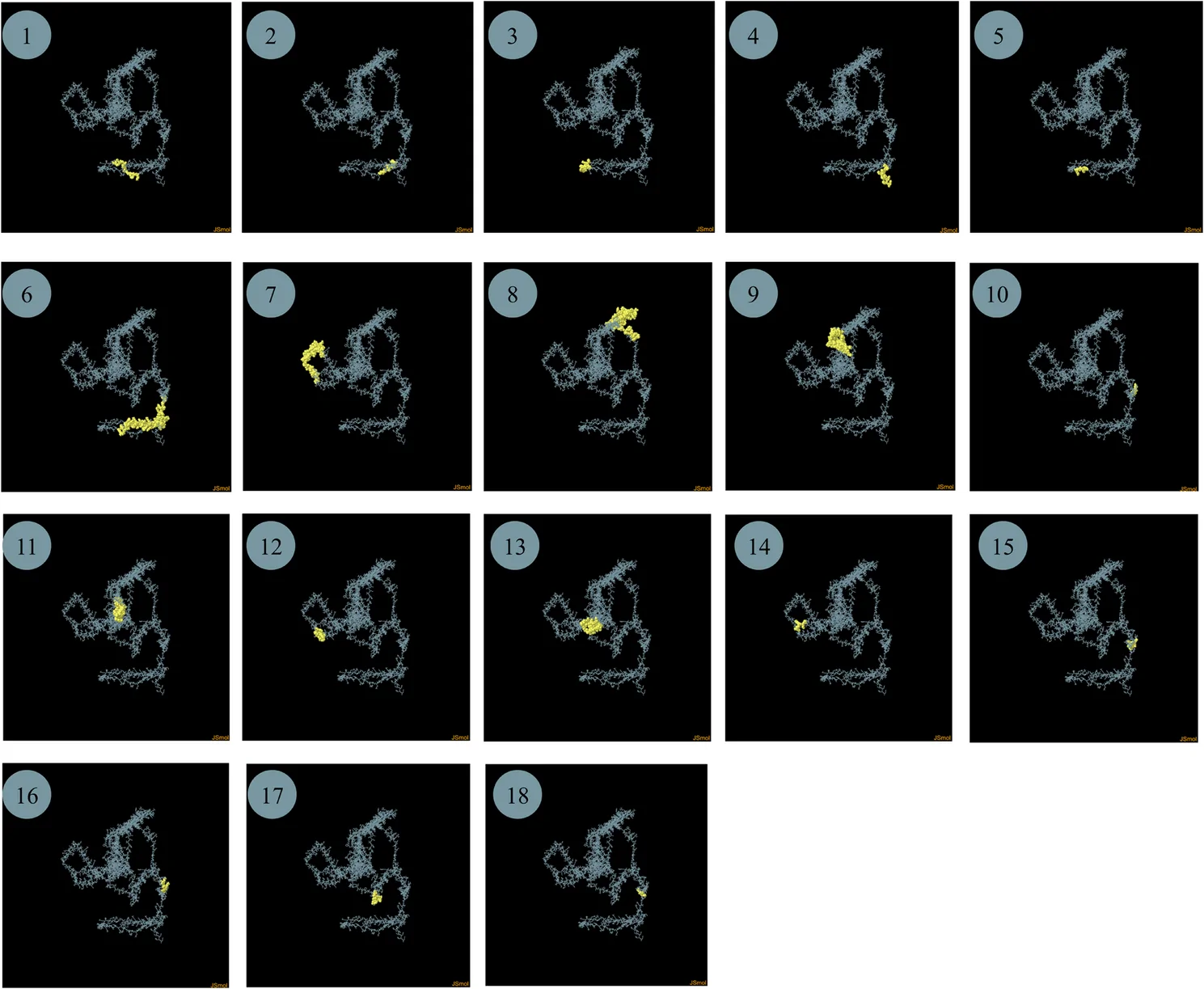
Analyzing discontinuous B-cell epitopes (1-18) The 3-dimensiional visualization of discontinuous B-cell epitopes. The yellow colour indicates the distribution of discontinuous B-cell epitopes in all sequences of the vaccine construct

The evaluation of discontinuous B-cell epitopes within the three-dimensional (3D) structure of the protein is essential for clarifying how the vaccine antigen may interact with antibodies in the host. These epitopes are antigenic regions that directly bind immune receptors, thereby contributing to immune activation. The analysis identified 18 discontinuous B-cell epitopes in the vaccine construct. All predicted epitopes had scores above 0.5, with the highest reaching 0.984. (Fig. 4) (Table S18). 

### Normal mode analysis of the multi-epitope vaccine construct

Interpretation of IMODS results is based on eigenvalue values that describe the degree of deformability of ligand and receptor bonds. The results showed the deformability of the vaccine construct binding with TLR2 (2.68 × 10 − 7) and with TLR4 (7.69 × 10 − 7). Indicated the multi-epitope vaccine construct binding with TLR had a lower eigenvalue than the multi-epitope vaccine construct with TLR2. (Figure S4.) 

Normal mode analysis (NMA) using the iMODS server was performed to evaluate the deformability and intrinsic flexibility of the vaccine–receptor complexes. The calculated eigenvalues were 2.68 × 10⁻⁷ for the vaccine–TLR2 complex and 7.69 × 10⁻⁷ for the vaccine–TLR4 complex, indicating differences in the energy required for deformation and intrinsic flexibility between the two complexes.

### Codon optimization and in-silico cloning of multi-epitope vaccines

**Fig. 5:**
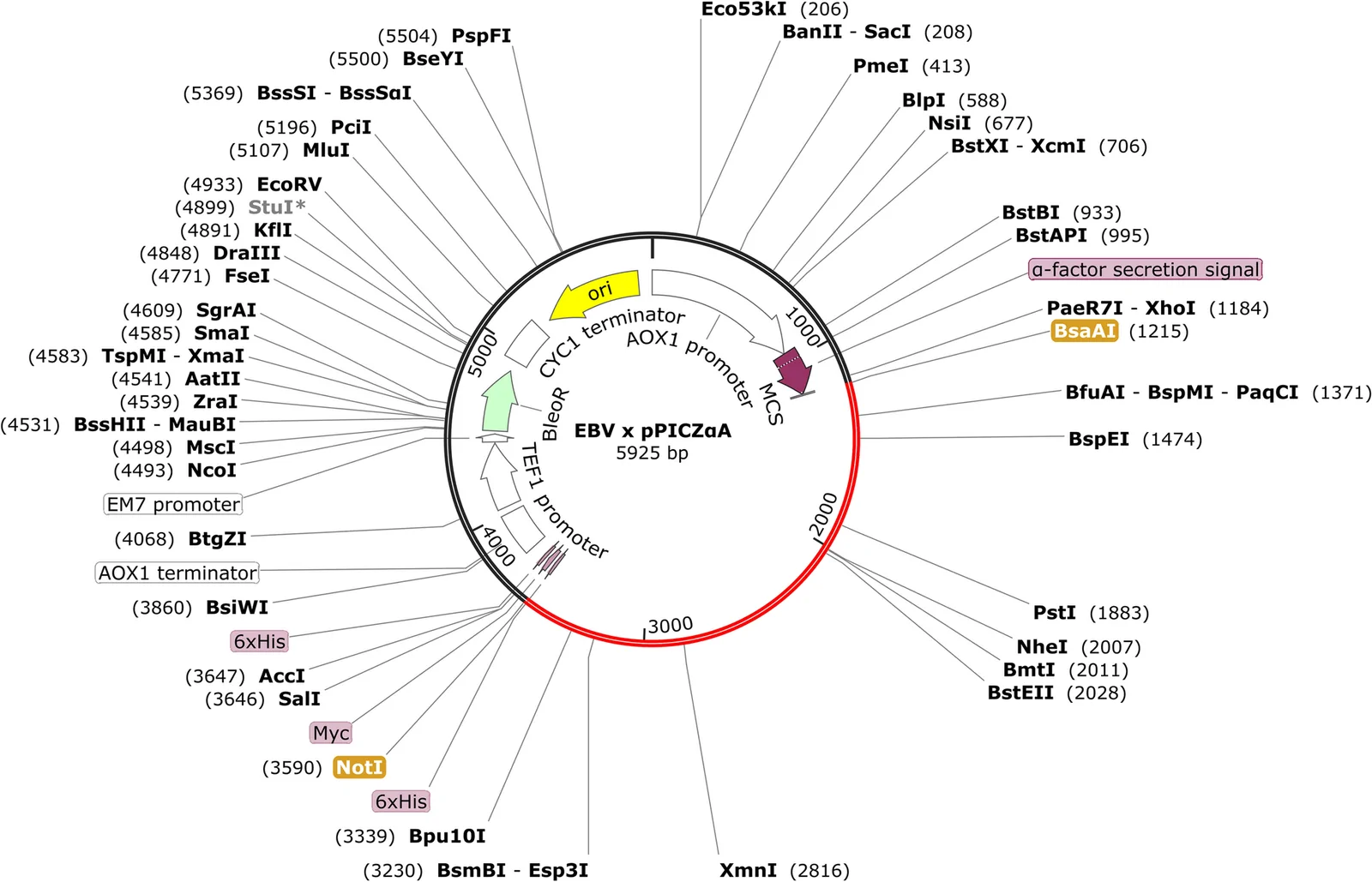
In silico cloning of the multi-epitope vaccine construct. The codon-optimized vaccine gene was inserted into the MCS of pPICZαA between BsaAI and NotI restriction sites under the AOX1 promoter for expression in Pichia pastoris. The recombinant plasmid (5925 bp) was generated using SnapGene v4.2

The codon optimization was performed using the IDT website to produce a gene sequence with *Pichia pastoris* as the organism of choice. The IDT results showed that the multi-epitope vaccine sequence had a complexity score of 13.2, which was classified as high complexity according to the platform’s criteria. This indicates that the optimized sequence was classified as relatively complex by the IDT platform; however, this computational classification alone does not establish its effect on recombinant protein expression. The optimized DNA sequence of the multi-epitope vaccine was subsequently used for in silico cloning using SnapGene, with pPICZαA as the cloning vector and the vaccine construct DNA sequence inserted between two restriction enzyme sites, namely BsaAI and NotI. (Fig. 5**).**

### Prediction of post-translational modification processes for vaccines

The predicted post-translational (PTM) modifications of the multi-epitope vaccine construct were predicted using the MusiteDeep website. The predicted PTMs were glycosylation (24), ubiquitination (14), phosphorylation (40), methylation (6), acetylation (7), and hydroxylation (9).

### Immune simulation

**Fig. 6:**
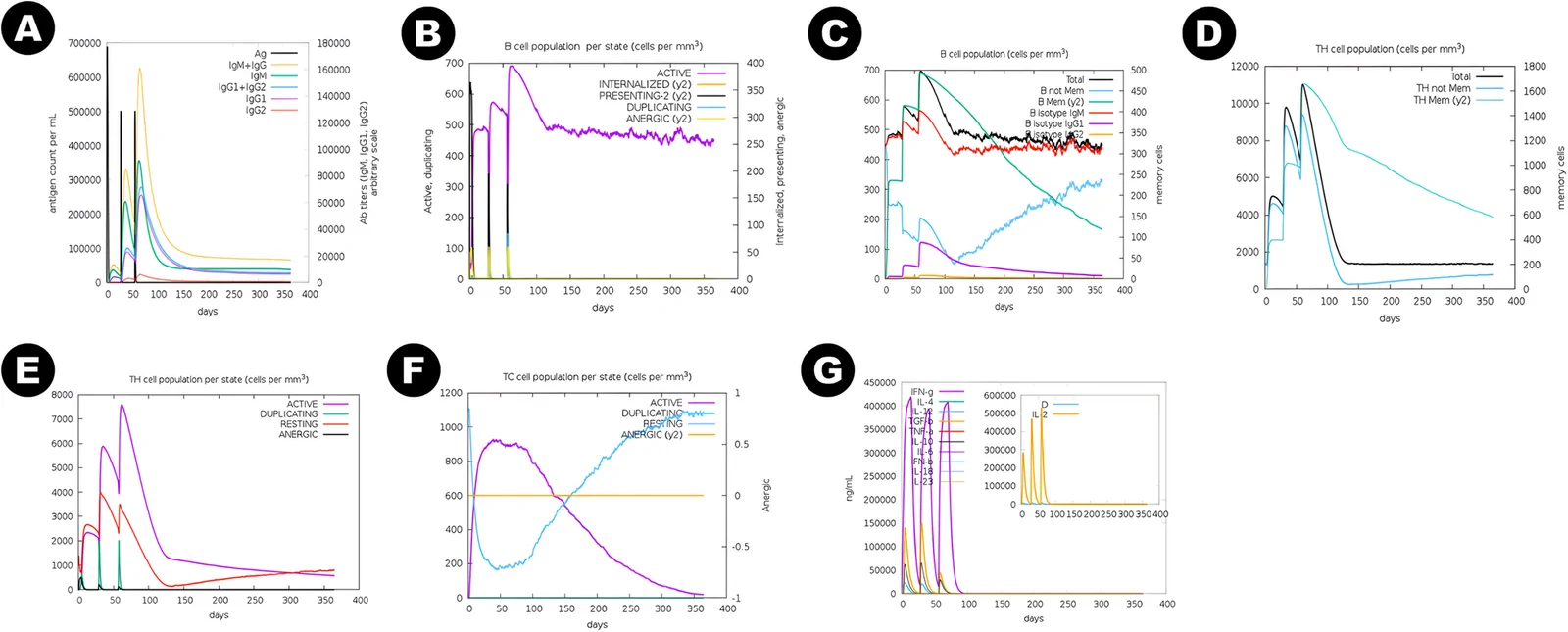
Immune stimulation after 3-dose antigen injections of the multi-epitope vaccine construct. Production of immunoglobulin; detailed subclasses are shown as colored peaks (**A**). Evolution of B-cell populations (**B**, **C**). T helper cell population over time (**D**, **E**). T-cytotoxic cell populations (**F**). Production of cytokines and interleukins (**G**)

To observe the development of cellular and humoral immune responses, an immune simulation was performed using the C-IMMSIM server. In this study, the experiment involved at least three injections at 28-day intervals. The immune simulation was conducted with all default parameters maintained, except for the injection timing, which was adjusted accordingly, and the number of simulation steps set to 1,095 (as this represents the conversion of 365 days in real life). Based on the simulation results, the immune response was effective, with high IgM concentrations followed by increases in immunoglobulins (IgG1 + IgG2, IgM, and IgG + IgM). Additionally, cytokine and interleukin production also increased (Fig. 6). 

## Discussion

The EBV protein selection was designed to address both prophylactic and tumor-targeted immune responses. gp350 was included as a prophylactic antigen due to its surface exposure on the viral envelope and its established role in inducing neutralizing antibodies that prevent EBV entry into host cells [2]. In contrast, EBNA1, LMP2A, and BZLF1 were selected as complementary EBV-derived antigenic targets based on their distinct biological roles in EBV persistence and reactivation. EBNA1 is particularly relevant to EBV-positive Burkitt lymphoma because it is the principal viral protein consistently expressed during latency I and is essential for maintenance of the viral episome. However, the glycine–alanine repeat (GAr) domain of EBNA1 can limit proteasomal processing and MHC class I presentation, representing an important mechanism of immune evasion. Nevertheless, EBNA1-derived peptides can be presented through MHC class II pathways and recognized by CD4⁺ T cells, supporting its relevance as a potential target for cellular immune responses[3, 5]. LMP2A was included as a complementary target because of its role in providing B-cell receptor-like survival signals independent of antigen engagement, primarily through activation of the Ras/PI3K/AKT pathway and destabilization of the cell-cycle inhibitor p27Kip1, which together enhance MYC-driven proliferation.6 Experimental evidence further indicates that LMP2A can allow MYC-driven tumors to arise without acquiring p53 mutations despite retaining an intact p53 pathway, suggesting a mechanism by which LMP2A cooperates with deregulated MYC to accelerate B-cell lymphomagenesis independently of classical p53 inactivation. However, LMP2A is not part of the canonical latency I expression program that defines classical BL, and its relevance is best supported in models of early or transitional EBV-driven B-cell transformation rather than in fully established latency I tumors, where its expression is often low and may be further limited by immune selection against LMP2A-expressing clones. LMP2A is therefore justified primarily as a therapeutic target for earlier or transitional stages of transformation, rather than for mature, latency I-restricted BL cells. ^5^ BZLF1, in contrast, is an immediate-early lytic protein and transcriptional activator that initiates the transition of EBV from latency to the lytic cycle by activating early viral genes involved in viral DNA replication and virion production. Although classical Burkitt lymphoma generally exhibits limited lytic gene expression, episodic or abortive lytic reactivation may provide transient exposure to lytic antigens and alter the antigenic landscape of EBV-infected cells. BZLF1 is also highly immunogenic and elicits robust CD4⁺ and CD8⁺ T-cell responses, making it a relevant antigen for broadening immune recognition of EBV-infected cells during lytic reactivation. Therefore, BZLF1 was included as a complementary antigen to extend immune coverage beyond the latency-associated antigen EBNA1 [1].

The selection of an appropriate construct necessitates careful evaluation of multiple essential aspects. In this study, epitope selection is not only based on antigenic, non-allergenic, non-toxic, and non-homologous criteria, but also on percentile scores representing high affinity with receptors. T-cell epitopes with the lowest percentile (≤ 2) show high affinity to CTL and HTL. CTLs play a role in adaptive immunity as antitumor effectors, recognizing epitopes presented by MHC-I and eliminating infected cells. These epitopes are generally 9 amino acids in length, recognized by MHC-I, and must exhibit a positive immunogenicity score to be considered immunogenic. HTL epitopes are generally 15 amino acids in length, recognized by MHC-II, and must be able to induce IFN-γ, IL-4, and IL-10; this is important for the adaptive immune system to fight cancer. While B-cell epitopes with scores above the selected threshold (≥ 0.51) were considered potential candidates for humoral immune responses. IgPred analysis was subsequently performed to predict their potential association with IgA, IgG, and IgE antibody respones [7, 11].

Each epitope used plays a crucial role during EBV infection. In this study, the vaccine constructs produced totaled 20, but only four met the criteria: antigenic, non-allergenic, non-toxic, soluble, with favourable physicochemical characteristics, and covering more than 50% of the population. The selected construct uses adjuvants. Adjuvants are additional components in vaccines that serve to increase the effectiveness of antigens in triggering the immune system. Adjuvants work by activating the PRRs (pattern recognition receptors) signaling pathway [20]. In this study, three adjuvants were used in the vaccine construction, namely OV ASP-1, 50 S ribosomal protein L7/L12, and HBD-3.

OV-ASP-1 is a protein capable of inducing T helper cells against various antigens. Through the activation of APCs, particularly dendritic cells (DC), via interaction with TLR-2 and TLR-4. This adjuvant is capable of inducing immunoglobulins (IgG1 and IgG2a) that enhance the immune response. Based on the results of predicting that the construction with the OV-ASP-1 adjuvant cannot be used because it is allergenic. This condition can trigger hypersensitivity reactions that affect the safety and tolerability of the vaccine. These reactions can occur in varying degrees of severity, ranging from mild local responses to more serious systemic reactions [21].

Human beta-defensin-3 is a protein that can be used as an adjuvant that plays a role in the immune response of epithelial barriers [22]. This role is relevant to the body’s defense mechanisms against infection, including the transmission of EBV. But construction of EBV multiepitope vaccines developed with hBdS-3 shows that none of these constructions has been fully established as an ideal vaccine candidate.

50 S ribosomal protein L7/L12 is used as an adjuvant due to its ability to induce the immune system and its role in DC-mediated tumor immunotherapy. This adjuvant, following Naveed et al. (2023), was used as an adjuvant for the construct, as it significantly improves recognition by TLRs and can polarize CD4^+^ cells. Its mechanism of action involves binding to TLR4, which subsequently triggers TLR signaling and activates both the MyD88- and TRIF-dependent pathways, resulting in DC activation. Once activated, DCs promote the stimulation of naive T lymphocytes, drive the polarization of CD4⁺ and CD8⁺ T cells, enhance IFN-γ secretion, and initiate cytotoxic responses mediated by T lymphocytes [23]. The results of the study show that four vaccine multi-epitope constructs (R2, R3, R4, and R5), except for the R1 construct, because its molecular weight was not within the 40–110 kDa range. Proteins with a molecular weight below 40 kDa don’t have enough epitopes to be efficiently recognized by the immune system, while proteins with a molecular weight above 110 kDa tend to be more difficult to express and purify. The construction of a multiepitope vaccine with the 50 S ribosomal L7/L12 adjuvant was then analyzed further.

Within the MHC-I antigen presentation pathway, the transporter associated with antigen processing (TAP) mediates the translocation of antigenic peptides into the endoplasmic reticulum, facilitating their subsequent loading onto MHC-I molecules [7]. In this study, the complete multi-epitope vaccine constructs were subjected to TAP-binding analysis to evaluate their predicted TAP-binding characteristics. The constructs showed comparable predicted TAP-binding profiles, suggesting no substantial difference in their predicted interaction with TAP. Similarly, secondary structure predictions using PSIPRED and SOPMA revealed no substantial differences between the constructs. In contrast, Trosetta analysis yielded a TM score > 0.5 for all construct non-favorable tertiary conformation. Subsequently, tertiary structure refinement was performed for all constructs to enhance overall model quality and improve resemblance to the native structure [24]. The GalaxyRefine server is capable of refining tertiary structures by generating 5 models. From these five models, the best model was selected based on GDT-HA, RMSD, MolProbity, Clash score, Poor rotamers, and Rama favored values. All five models were evaluated with PROSA, ERRAT, and PROCHECK to determine the quality of the tertiary structure refinement results of the multi-epitope vaccine construct model. The quality of the multi-epitope vaccine construct was evaluated using PROCHECK, PROSA, and ERRAT to assess the accuracy and quality of the protein structure, while CABSFLEX was used to predict flexibility. The overall results showed that the vaccine model had good structural quality and adequate stability to support molecular interactions.

In this study, molecular docking analysis was performed to explore the potential interaction between the vaccine constructs and TLR2 and TLR4 using ClusPro 2.0 and HDOCK. ClusPro 2.0 ranking was based on predicted binding energy, whereas HDOCK results were interpreted according to confidence scores. The R5 construct demonstrated comparatively favorable docking scores toward both TLR2 and TLR4 across the two platforms. TLR2 and TLR4 are surface-expressed pattern-recognition receptors implicated in EBV pathogenesis. TLR2 has been reported to recognize EBV glycoprotein gp350 and promote B-cell activation, leading to the release of monocyte chemotactic protein-1 (MCP-1) from macrophages. In contrast, TLR4 activation contributes to antiviral immune responses by inducing IFN-α production, which in turn induces IL-27 expression and supports T helper cell responses [13]. Nevertheless, the docking findings in this study only indicate a potential structural compatibility between the vaccine construct and these receptors. Experimental validation is required to determine whether the predicted interactions translate into functional immune activation in vitro or in vivo.

To proceed with molecular dynamics prediction, only the best construct among the four candidates was selected. All constructs exhibited antigenic, non-allergenic, non-toxic properties, as well as favorable physicochemical characteristics. However, based on tertiary structure prediction and binding affinity to TLR2 and TLR4 receptors, the R5 construct was identified as the most promising candidate for development as a prophylactic and therapeutic multi-epitope vaccine against EBV-positive Burkitt lymphoma. Molecular dynamics simulation was performed to evaluate the stability of the R5 vaccine construct in complex with TLR2 and TLR4 receptors. This focused on the deformability level, determined by the eigenvalue, which reflects the energy required for conformational changes. The eigenvalue also indicates the interaction between the ligand and receptor, where a low eigenvalue indicates good stability and flexibility of the molecular motion of the docked protein complex [25]. The predicted receptor interaction reflects the structural behavior of the complete multi-epitope vaccine construct. Because docking was performed using the full construct, the relative contribution of the adjuvant, linkers, and EBV-derived epitopes to receptor binding could not be determined independently. Thus, the observed interaction should be interpreted as a property of the assembled vaccine structure rather than as evidence that a specific component predominantly drives receptor binding.

In this study, in silico cloning was performed using an expression system with Pichia pastoris and the pPICZαA vector, which had previously undergone codon optimization to increase protein expression in specific host organisms by adjusting the codon sequence. The pPICZαA vector contains the AOX1 promoter, which enables high and tightly regulated protein expression. Previous studies have demonstrated that pPICZαA allows expression of gp350 and LMP1 in yeast, resulting in strong immunogenicity [26].

The construction of a multiepitope vaccine was assessed by PTM, which showed that phosphorylation was necessary for a total of 40 phosphorylation sites, 24 glycosylation sites, 14 ubiquitination sites, 9 hydroxylation sites, 7 acetylation sites, and 6 methylation sites. Phosphorylation was the most frequently predicted modification and may influence antigen processing and immune recognition. However, these predictions do not necessarily indicate that the same modifications will occur in recombinant protein expressed in *Pichia pastoris*, as yeast and human cells differ in their modifying enzymes and substrate specificities [27].

Glycosylation was the second most frequently predicted modification and is particularly relevant to *P. pastoris* expression. Yeast-specific glycosylation may differ from native human or EBV glycosylation and could affect protein folding, antigen stability, and epitope accessibility. Therefore, the predicted glycosylation sites may potentially influence immune recognition and should be experimentally characterized to determine the actual glycan profile of the expressed construct [28].

Post-translational modification analysis identified potential sites for phosphorylation, glycosylation, ubiquitination, methylation, acetylation, and hydroxylation. These modifications may influence protein folding, stability, immune processing, and degradation. In the context of Pichia pastoris expression, the predicted glycosylation pattern is particularly relevant because yeast-specific glycosylation may affect antigen structure and immunogenicity, while other modifications may influence protein stability and intracellular processing. However, these predictions are computational and do not establish that the modifications will occur experimentally or adversely affect vaccine safety [28]. Finally, the R5 vaccine construct was simulated with injections administered at intervals of 0, 28, and 56 days, for a total of three doses. The simulation predicted the induction of both cellular and humoral immune responses, suggesting that the construct may have immunological potential against EBV-associated antigens. However, these findings are based solely on computational simulation and do not establish protective efficacy against EBV infection or antitumor activity against Burkitt lymphoma.

Despite these findings, this study has an important limitation, as the proposed R5 multi-epitope vaccine was evaluated exclusively using in silico approaches and therefore its predicted antigenicity, immunogenicity, and potential antitumor activity remain experimentally unverified. In addition, computational predictions cannot fully capture the complexity of antigen processing, immune-cell interactions, and host responses in biological systems. Future studies should therefore prioritize experimental validation of the proposed R5 multi-epitope vaccine through a stepwise preclinical development strategy. The initial stage should involve synthesis, cloning, and sequence verification of the R5 construct, followed by heterologous expression in Pichia pastoris. The recombinant protein should then be purified and characterized for molecular integrity, purity, solubility, and structural properties. Subsequent in vitro studies should evaluate its antigenicity and immunogenicity through antigen-specific antibody binding, dendritic-cell activation, cytokine production, and T-cell responses using appropriate PBMC-based assays. Functional validation should further determine whether vaccine-induced immune responses can recognize EBV-positive malignant B cells, using established Burkitt lymphoma models such as Raji cells. Finally, in vivo studies should assess immunogenicity, durability of the immune response, tolerability, and preliminary safety, while providing initial evidence of protective or antitumor activity.

## Conclusion

The R5 multi-epitope vaccine construct, incorporating the 50 S ribosomal L7/L12 adjuvant, exhibits favorable characteristics regarding antigenicity, allergenicity, physicochemical and structural properties, as well as predicted interactions with TLR2 and TLR4. Further immune simulations predict the induction of both cellular and humoral immune responses following a simulated three-dose regimen administered at 28-day intervals. These computational findings indicate that the R5 construct warrants further investigation as a vaccine candidate targeting EBV-associated antigens in the context of Burkitt lymphoma. However, the current in silico findings do not establish prophylactic or therapeutic efficacy against EBV infection or Burkitt lymphoma. Experimental validation through in vitro and in vivo studies is required to evaluate its immunogenicity, safety, antigen processing, and potential protective or antitumor activity. .

## Supplementary Information

Supplementary Material 1.

## Data Availability

No datasets were generated or analysed during the current study.

## Abbreviations

EBV: Epstein-Barr Virus
BL: Burkitt lymphoma
CTL: Cytotoxic T cells
HTL: Helper T cells
MHC-I: Major Histocompatibility Complex Class-I
MHC-II: Major Histocompatibility Complex Class-II
HLA: Human Leukocyte Antigen
TLR: Toll-Like Receptor
PTM: Post-Translational Modifications
