# Supplementary material for: Adjuvant selection and optimization in the design of an epstein–barr virus–based multi-epitope vaccine against Burkitt lymphoma
Source: J Egypt Natl Canc Inst. 2026 Oct 27;38:82. doi: 10.1186/s43046-026-00423-1 (PMC13710498; doi:10.1186/s43046-026-00423-1)
Supplement: Supplementary file 1 [file 43046_2026_423_MOESM1_ESM.docx]

**Supplementary Material**

**Table S1.** Websites utilized in this research

| NCBI virus website | <https://www.ncbi.nlm.nih.gov/> |
| --- | --- |
| Vaxijen v2.0 website | <https://ddgpharmfac.net/vaxijen/VaxiJen/VaxiJen.html)> |
| ProtParam website | <https://web.expasy.org/protparam/> |
| IEDB website | <http://tools.iedb.org/mhci/> |
| ABCpred website | <http://crdd.osdd.net/raghava/abcpred/> |
| Algpred 2.0 website | <https://webs.iiitd.edu.in/raghava/algpred2/algo.html> |
| ToxinPred website | [https://webs.iiitd.edu.in/raghava/toxinpred/protein.php](https://webs.iiitd.edu.in/raghava/toxinpred/protein.php)) |
| NCBI BLAST website | <https://blast.ncbi.nlm.nih.gov/Blast.cgi> |
| IEDB Population Coverage website | <http://tools.iedb.org/population/> |
| SOLpro website | <http://scratch.proteomics.ics.uci.edu> |
| TAPPred website | <https://webs.iiitd.edu.in/raghava/tappred/> |
| PSIPRED website | <http://bioinf.cs.ucl.ac.uk/psipred/> |
| SOPMA website | <https://npsa-prabi.ibcp.fr/cgi-bin/npsa_automat.pl?page=/NPSA/npsa_sopma.html> |
| trRosetta website | <https://yanglab.nankai.edu.cn/trRosetta/> |
| GalaxyRefine website | <http://galaxy.seoklab.org/cgi-bin/submit.cgi?type=REFINE> |
| Ellipro | <https://tools.iedb.org/ellipro/> |
| ProSA website | <https://prosa.services.came.sbg.ac.at/prosa.php> |
| ERRAT website | <https://saves.mbi.ucla.edu/> |
| PROCHECK website | <https://saves.mbi.ucla.edu/> |
| CABSFLEX 2.0 website | <http://biocomp.chem.uw.edu.pl/CABSflex2/submit> |
| RSCB PDB website | [https://www.rcsb.org/](https://www.rcsb.org/)) |
| ClusPro website | <https://cluspro.bu.edu/home.php> |
| HDOCK website | <http://hdock.phys.hust.edu.cn/> |
| iMODS website | <http://imods.chaconlab.org> |
| IDT website | <https://www.idtdna.com/pages/tools/codon-optimizationtool> |
| SnapGene v4.2 website | <https://www.snapgene.com/> |
| MusiteDeep website | <https://www.musite.net/> |
| C-IMMSIM website | <https://kraken.iac.rm.cnr.it/C-IMMSIM/index.php?page=1> |

**Table S2**. CTL epitopes gp350 of EBV

| **No** | **Allele** | **Peptide** | **Start-End** | **Percentile**  **score** | **Antigenic** | **Allergenic** | **Toxic** | **Immunogenic** | **Homologous** |
| --- | --- | --- | --- | --- | --- | --- | --- | --- | --- |
| 1. | HLA-A*30:01 | KTLIITRTA | 401-409 | 0.11 | Yes | No | No | Yes (0.36282) | No |
|  | HLA-B*57:01 |  |  | 1.6 |  |  |  |  |  |
|  | HLA-A*32:01 |  |  | 1.7 |  |  |  |  |  |
|  | HLA-B*58:01 |  |  | 1.8 |  |  |  |  |  |
|  | HLA-A*30:02 |  |  | 1.9 |  |  |  |  |  |
|  | HLA-A*02:06 |  |  | 2 |  |  |  |  |  |
| 2. | HLA-A*68:01 | STNITAVVR | 167-175 | 0.13 | Yes | No | No | Yes (0.26541) | No |
|  | HLA-A*31:01 |  |  | 0.14 |  |  |  |  |  |
|  | HLA-A*11:01 |  |  | 0.3 |  |  |  |  |  |
|  | HLA-A*33:01 |  |  | 0.33 |  |  |  |  |  |
|  | HLA-A*03:01 |  |  | 1.3 |  |  |  |  |  |
|  | HLA-A*30:01 |  |  | 1.3 |  |  |  |  |  |
| 3. | HLA-A*68:02 | NSTNITAVV | 166-174 | 0.15 | Yes | No | No | Yes (0.22937) | No |
|  | HLA-B*51:01 |  |  | 1.1 |  |  |  |  |  |
| 4. | HLA-B*35:01 | MPTNTTDIT | 315-323 | 1.4 | Yes | No | No | Yes (0.17691) | No |
| 5. | HLA-B*51:01 | FASNRTFDI | 383–391 | 0.95 | Yes | No | No | Yes (0.13849) | No |
|  | HLA-B*08:01 |  |  | 2 |  |  |  |  |  |
| 6. | HLA-A*32:01 | RAQGLDVTL | 175–183 | 0.62 | Yes | No | No | Yes (0.06410) | No |
|  | HLA-B*58:01 |  |  | 0.64 |  |  |  |  |  |
|  | HLA-B*07:02 |  |  | 0.88 |  |  |  |  |  |
|  | HLA-B*57:01 |  |  | 1.4 |  |  |  |  |  |
|  | HLA-A*02:06 |  |  | 1.5 |  |  |  |  |  |
|  | HLA-B*35:01 |  |  | 1.6 |  |  |  |  |  |
|  | HLA-B*53:01 |  |  | 1.8 |  |  |  |  |  |
|  | HLA-B*40:01 |  |  | 1.8 |  |  |  |  |  |
| 7. | HLA-B*08:01 | MLVLQWASL | 861–869 | 0.53 | Yes | No | No | Yes (0.03431) | No |
|  | HLA-A*02:01 |  |  | 1.6 |  |  |  |  |  |
|  | HLA-A*02:03 |  |  | 1.7 |  |  |  |  |  |
|  | HLA-A*32:01 |  |  | 1.7 |  |  |  |  |  |
| 8. | HLA-B*51:01 | WASLAVLTL | 866–874 | 0.47 | Yes | No | No | Yes (0.02542) | No |
|  | HLA-B*35:01 |  |  | 0.62 |  |  |  |  |  |
|  | HLA-B*53:01 |  |  | 0.77 |  |  |  |  |  |
|  | HLA-B*08:01 |  |  | 1.7 |  |  |  |  |  |

HLA, Human Leukocyte Antigen; In bold, epitopes selected for the multi-epitope vaccine construct

**Table S3.** CTL epitopes of EBNA1 EBV

| **No** | **Allele** | **Peptide** | **Start-End** | **Percentile**  **score** | **Antigenic** | **Allergenic** | **Toxic** | **Immunogenic** | **Homologous** |
| --- | --- | --- | --- | --- | --- | --- | --- | --- | --- |
| 1. | HLA-A*02:06 | RTTDEGTWV | 494-503 | 1.6 | Yes | No | No | Yes  (0.32421) | No |
| 2. | HLA-A*30:01 | RKKGGWFGK | 457-466 | 0.51 | Yes | No | No | Yes  (0.3265) | No |

HLA, Human Leukocyte Antigen

**Table S4**. CTL epitopes LMP2A of EBV

| **No** | **Allele** | **Peptide** | **Start-End** | **Percentile**  **score** | **Antigenic** | **Allergenic** | **Toxic** | **Immunogenic** | **Homologous** |
| --- | --- | --- | --- | --- | --- | --- | --- | --- | --- |
| 1. | HLA-A*02:03 | YLFWLAAIA | 132–140 | 1.2 | Yes | No | No | Yes (0.39770) | No |
|  | HLA-A*02:01 |  |  | 1.4 |  |  |  |  |  |
| 2. | HLA-A*23:01 | PYLFWLAAI | 131–139 | 0.45 | Yes | No | No | Yes (0.37534) | No |
|  | HLA-A*24:02 |  |  | 0.53 |  |  |  |  |  |
| 3. | HLA-A*02:03 | FLIFLIGFA | 457–465 | 1.1 | Yes | No | No | Yes (0.37248) | No |
|  | HLA-A*02:01 |  |  | 1.9 |  |  |  |  |  |
| 4. | HLA-B*07:02 | APYLFWLAA | 130–138 | 0.78 | Yes | No | No | Yes(0.32365) | No |
|  | HLA-B*51:01 |  |  | 1.6 |  |  |  |  |  |
| 5. | HLA-A*02:01 | MLLWTLVVL | 328–336 | 0.61 | Yes | No | No | Yes (0.30561) | No |
|  | HLA-A*32:01 |  |  | 1.3 |  |  |  |  |  |
|  | HLA-A*02:06 |  |  | 1.6 |  |  |  |  |  |
|  | HLA-A*02:03 |  |  | 1.8 |  |  |  |  |  |
| 6. | HLA-A*31:01 | ILAYRRRWR | 232–240 | 0.89 | Yes | No | No | Yes (0.28120) | Tidak |
|  | HLA-A*33:01 |  |  | 1.1 |  |  |  |  |  |
| 7. | HLA-A*02:06 | LIGFALFGV | 461–469 | 1.5 | Yes | No | No | Yes (0.27506) | Tidak |
|  | HLA-A*02:01 |  |  | 1.5 |  |  |  |  |  |
| 8. | HLA-A*68:02 | VAPYLFWLA | 129–137 | 2 | Yes | No | No | Yes (0.27254) | No |
| 9. | HLA-A*33:01 | GFALFGVIR | 463–471 | 0.92 | Yes | No | No | Yes (0.26004) | No |
|  | HLA-A*31:01 |  |  | 1.1 |  |  |  |  |  |
| 10. | HLA-A*24:02 | TWRIEDPPF | 197–205 | 0.59 | Yes | No | No | Yes (0.25326) | No |
|  | HLA-A*23:01 |  |  | 0.61 |  |  |  |  |  |
| 11. | HLA-A*02:01 | LMLLWTLVV | 327–335 | 1.3 | Yes | No | No | Yes (0.25224) | No |
| 12. | HLA-A*33:01 | FFAICLTWR | 191–199 | 0.69 | Yes | No | No | Yes(0.24586) | No |
| 13. | HLA-A*26:01 | CLPVIVAPY | 124–132 | 0.9 | Yes | No | No | Yes(0.23294) | No |
|  | HLA-B*15:01 |  |  | 1.6 |  |  |  |  |  |
| 14. | HLA-B*51:01 | FAICLTWRI | 192–200 | 0.61 | Yes | No | No | Yes (0.23187) | No |
|  | HLA-A*68:02 |  |  | 0.78 |  |  |  |  |  |
|  | HLA-A*02:06 |  |  | 1 |  |  |  |  |  |
| 15. | HLA-B*51:01 | IGFALFGVI | 462–470 | 1.4 | Yes | No | No | Yes (0.22949) | No |
| 16. | HLA-A*02:01 | MLLLIVAGI | 395–403 | 1.4 | Yes | No | No | Yes (0.20652) | No |
|  | HLA-A*02:03 |  |  | 1.9 |  |  |  |  |  |
| 17. | HLA-A*24:02 | AWILTAGFL | 450–458 | 1.7 | Yes | No | No | Yes (0.20367) | No |
|  | HLA-A*23:01 |  |  | 1.8 |  |  |  |  |  |
| 18. | HLA-A*31:01 | VLLILAYRR | 229–237 | 0.56 | Yes | No | No | Yes (0.18347) | No |
|  | HLA-A*33:01 |  |  | 0.76 |  |  |  |  |  |
| 19. | HLA-A*68:02 | STVVTATGL | 149–157 | 0.38 | Yes | No | No | Yes (0.18213) | No |
|  | HLA-A*26:01 |  |  | 1.6 |  |  |  |  |  |
| 20. | HLA-A*23:01 | TFFAICLTW | 190–198 | 0.15 | Yes | No | No | Yes (0.16954) | No |
|  | HLA-A*24:02 |  |  | 0.28 |  |  |  |  |  |
|  | HLA-A*32:01 |  |  | 0.56 |  |  |  |  |  |
|  | HLA-B*53:01 |  |  | 0.78 |  |  |  |  |  |
|  | HLA-B*58:01 |  |  | 0.83 |  |  |  |  |  |
|  | HLA-B*57:01 |  |  | 0.96 |  |  |  |  |  |
| 21. | HLA-A*02:01 | LLWTLVVLL | 329–337 | 0.26 | Yes | No | No | Yes(0.16738) | No |
|  | HLA-A*02:06 |  |  | 0.84 |  |  |  |  |  |
|  | HLA-A*02:03 |  |  | 1.5 |  |  |  |  |  |
| 22. | HLA-A*02:01 | PLLGAVTVV | 267–275 | 1.5 | Yes | No | No | Yes (0.16434) | No |
| 23. | HLA-B*58:01 | LLLLAFVLW | 279–287 | 0.56 | Yes | No | No | Yes(0.16190) | No |
|  | HLA-B*57:01 |  |  | 0.58 |  |  |  |  |  |
|  | HLA-A*32:01 |  |  | 0.62 |  |  |  |  |  |
|  | HLA-A*23:01 |  |  | 1.4 |  |  |  |  |  |
|  | HLA-B*53:01 |  |  | 1.7 |  |  |  |  |  |
| 24. | HLA-A*02:01 | LLMLLWTLV | 326–334 | 1.4 | Yes | No | No | Yes (0.15583) | No |
|  | HLA-A*02:03 |  |  | 1.9 |  |  |  |  |  |
| 25. | HLA-A*02:01 | SLLFALLAA | 207–215 | 0.72 | Yes | No | No | Yes (0.15536) | No |
|  | HLA-A*02:03 |  |  | 0.99 |  |  |  |  |  |
|  | HLA-A*02:06 |  |  | 1.1 |  |  |  |  |  |
| 26. | HLA-B*57:01 | LILAYRRRW | 231–239 | 0.23 | Yes | No | No | Yes (0.15481) | No |
|  | HLA-A*32:01 |  |  | 0.29 |  |  |  |  |  |
|  | HLA-B*58:01 |  |  | 0.42 |  |  |  |  |  |
|  | HLA-B*53:01 |  |  | 1.9 |  |  |  |  |  |
| 27. | HLA-A*02:01 | GLQGIYVLV | 218–226 | 0.1 | Yes | No | No | Yes (0.15098) | No |
|  | HLA-A*02:03 |  |  | 0.13 |  |  |  |  |  |
|  | HLA-A*02:06 |  |  | 0.28 |  |  |  |  |  |

HLA, Human Leukocyte Antigen; In bold, epitopes selected for the multi-epitope vaccine construct

**Table S5.** CTL Epitopes of BZLF1 EBV

| No | Allele | Peptide | Start-End | Percentile score | Antigenic | Allergenic | Toxic | Immunogenic | Homologous |
| --- | --- | --- | --- | --- | --- | --- | --- | --- | --- |
| 1 | HLA-A*30:01 | HYREVASAK | 199-207 | 0.06 | Yes | No | No | Yes (0.07782) | No |
|  | HLA-A*33:01 |  |  | 0.35 |  |  |  |  |  |
|  | HLA-A*31:01 |  |  | 0.66 |  |  |  |  |  |
|  | HLA-A*03:01 |  |  | 2 |  |  |  |  |  |
| 2 | HLA-B*44:02 | SELEIKRYK | 173-181 | 0.51 | Yes | No | No | Yes (0.06527) | No |
|  | HLA-B*44:03 |  |  | 0.52 |  |  |  |  |  |
|  | HLA-A*11:01 |  |  | 2 |  |  |  |  |  |

HLA, Human Leukocyte Antigen

**Table S6**. HTL epitopes gp350 of EBV

| **No** | **Allele** | **Peptide** | **Start-End** | **Percentile score** | **Antigenic** | **Allergenic** | **Toxic** | **IFN- γ** | **IL-4** | **IL-10** | **Homologous** |
| --- | --- | --- | --- | --- | --- | --- | --- | --- | --- | --- | --- |
| 1. | HLA-DRB4*01:01 | TAVVRAQGLDVTLPL | 171-185 | 0.08 | Yes | No | No | + | - | - | No |
| 2. | HLA-DRB1*07:01 | ENITQVTPASISTHH | 700-714 | 1.4 | Yes | No | No | + | - | - | No |
| 3. | HLA-  DRB1*08:02 | QDSNFSVKTEMLGNE | 192-206 | 0.53 | Yes | No | No | + | - | - | No |
|  | HLA-  DQA1*04:01/DQB1*04:02 |  |  | 1.7 |  |  |  |  |  |  |  |
| 4. | HLA-DRB1*08:02 | DSNFSVKTEMLGNEI | 193-207 | 0.56 | Yes | No | No | + | - | - | No |
| 5. | HLA-DQA1*04:01/DQB1*04:02 | VTPASISTHHVSTSS | 705-719 | 1.5 | Yes | No | No | + | - | - | No |
| 6. | HLA-DRB1*08:02 | AQDSNFSVKTEMLGN | 191-205 | 1,6 | Yes | No | No | + | - | - | No |

HLA, Human Leukocyte Antigen; IFN, interferon; IL, interleukin.

**Table S7**. HTL epitopes of EBNA1 EBV

| **No** | **Allele** | **Peptide** | **Start-End** | **Percentile**  **score** | **Antigenic** | **Allergenic** | **Toxic** | **IFN- γ** | **IL-4** | **IL-10** | **Homologous** |
| --- | --- | --- | --- | --- | --- | --- | --- | --- | --- | --- | --- |
| 1. | HLA-DRB1*11:01 | KGGWFGKHRGQGGSN | 460-474 | 0.82 | Yes | No | No | + | - | - | No |
|  | HLA-DRB1*13:02 |  |  | 2 |  |  |  |  |  |  |  |
| 2. | HLA-DRB1*11:01 | KTSLYNLRRGTALAI | 513-527 | 0.99 | Yes | No | No | + | - | - | No |

HLA, Human Leukocyte Antigen; IFN, interferon; IL, interleukin.

**Table S8**. HTL epitopes LMP2A of EBV

| **No** | **Allele** | **Peptide** | **Start-End** | **Percentile**  **score** | **Antigenic** | **Allergenic** | **Toxic** | **IFN- γ** | **IL-4** | **IL-10** | **Homologous** |
| --- | --- | --- | --- | --- | --- | --- | --- | --- | --- | --- | --- |
| 1. | HLA-DPA1*02:01/DPB1*01:01 | QTNFKSLSSTEFIPN | 377-391 | 0.62 | Yes | No | No | + | + | - | No |
|  | HLA-DRB1*01:01 |  |  | 1.1 |  |  |  |  |  |  |  |
|  | HLA-DRB1*07:01 |  |  | 1.3 |  |  |  |  |  |  |  |
|  | HLA-DPA1*01:03/DPB1*04:01 |  |  | 1.4 |  |  |  |  |  |  |  |
|  | HLA-DRB1*12:01 |  |  | 1.5 |  |  |  |  |  |  |  |
|  | HLA-DPA1*03:01/DPB1*04:02 |  |  | 1.8 |  |  |  |  |  |  |  |
| 2. | HLA-DPA1*02:01/DPB1*01:01 | FKSLSSTEFIPNLFC | 380-394 | 0.72 | Yes | No | No | - | + | - | No |
|  | HLA-DPA1*01:03/DPB1*04:01 |  |  | 0.85 |  |  |  |  |  |  |  |
|  | HLA-DPA1*03:01/DPB1*04:02 |  |  | 1.8 |  |  |  |  |  |  |  |
| 3. | HLA-DPA1*02:01/DPB1*01:01 | KSLSSTEFIPNLFCM | 381-395 | 1.8 | Yes | No | No | - | + | - | No |
|  | HLA-DPA1*01:03/DPB1*04:01 |  |  | 1.9 |  |  |  |  |  |  |  |
| 4. | HLA-DPA1*01:03/DPB1*02:01 | GGSILQTNFKSLSST | 372-386 | 0.15 | Yes | No | No | + | - | - | No |
|  | HLA-DPA1*01:03/DPB1*04:01 |  |  | 0.28 |  |  |  |  |  |  |  |
|  | HLA-DPA1*03:01/DPB1*04:02 |  |  | 0.31 |  |  |  |  |  |  |  |
|  | HLA-DPA1*02:01/DPB1*01:01 |  |  | 0.41 |  |  |  |  |  |  |  |
|  | HLA-DPA1*02:01/DPB1*05:01 |  |  | 1.1 |  |  |  |  |  |  |  |
| 5. | HLA-DPA1*01:03/DPB1*02:01 | AGGSILQTNFKSLSS | 371-385 | 0.2 | Yes | No | No | + | - | - | No |
|  | HLA-DPA1*01:03/DPB1*04:01 |  |  | 0.35 |  |  |  |  |  |  |  |
|  | HLA-DPA1*03:01/DPB1*04:02 |  |  | 0.39 |  |  |  |  |  |  |  |
|  | HLA-DPA1*02:01/DPB1*01:01 |  |  | 0.46 |  |  |  |  |  |  |  |
|  | HLA-DPA1*02:01/DPB1*05:01 |  |  | 1.6 |  |  |  |  |  |  |  |

HLA, Human Leukocyte Antigen; IFN, interferon; IL, interleukin; In bold: epitopes selected for the multi-epitope vaccine construct.

**Table S9**. HTL epitopes BZLF1

| No | Allele | Peptide | Start- End | Percentile  score | Antigenic | Allergenic | Toxic | IFN- γ | IL-4 | IL-10 | Homologous |
| --- | --- | --- | --- | --- | --- | --- | --- | --- | --- | --- | --- |
| 1. | HLA-DQA1*05:01/DQB1*03:01 | HYREVASAKSSENDR | 199-213 | 0,43 | Yes | No | No | + | - | + | No |
|  | HLA-DPA1*02:01/DPB1*14:01 |  |  | 0,77 |  |  |  |  |  |  |  |
| 2. | HLA-DQA1*05:01/DQB1*03:01 | YREVASAKSSENDRL | 200-214 | 0,43 | Yes | No | No | + | - | + | No |
| 3. | HLA-DRB1*15:01 | SELEIKRYKNRVASR | 173-187 | 0,02 | Yes | No | No | + | + | + | No |
|  | HLA-DQA1*04:01/DQB1*04:02 |  |  | 0,68 |  |  |  |  |  |  |  |
|  | HLA-DQA1*01:01/DQB1*05:01 |  |  | 1,6 |  |  |  |  |  |  |  |
| 4. | HLA-DRB1*15:01 | DSELEIKRYKNRVAS | 172-186 | 0,03 | Yes | No | No | + | + | + | No |
| 5. | HLA-DRB1*04:01 | HLLQHYREVASAKSS | 195-209 | 0,68 | Yes | No | No | - | - | + | No |
| 6. | HLA-DRB5*01:01 | CRAKFKHLLQHYREV | 189-203 | 0,77 | Yes | No | No | - | + | + | No |
|  | HLA-DPA1*02:01/DPB1*05:01 |  |  | 2 |  |  |  |  |  |  |  |
| 7. | HLA-DRB5*01:01 | KCRAKFKHLLQHYRE | 188-202 | 1,2 | Yes | No | No | - | + | + | No |
| 8. | HLA-DRB1*15:01 | FKHLLQHYREVASAK | 193-207 | 1,3 | Yes | No | No | - | + | + | No |

HLA, Human Leukocyte Antigen; IFN, interferon; IL, interleukin.

**Table S10**. B-cell epitopes of EBNA1, LMP2A, and BZLF1 EBV

| **Protein** | **No** | **Peptide** | **Percentile score** | **Antigenic** | **Allergen** | **Toxic** | **Ig** | **Homologous** |
| --- | --- | --- | --- | --- | --- | --- | --- | --- |
| EBNA1 | 1. | GAPGGSGSGPRHRDGV | 0.87 | Yes | No | No | IgG | No |
|  | 2. | RGRGRGEKRPRSPSSQ | 0.87 | Yes | No | No | IgG | No |
|  | 3. | GRGEKRPRSPSSQSSS | 0.8 | Yes | No | No | IgG | No |
|  | 4. | GGRPGAPGGSGSGPRH | 0.77 | Yes | No | No | IgG | No |
|  | 5. | KKGGWFGKHRGQGGSN | 0.73 | Yes | No | No | IgG | No |
|  | 6. | RPGAPGGSGSGPRHRD | 0.62 | Yes | No | No | IgG | No |
|  | 7. | PGAPGGSGSGPRHRDG | 0.58 | Yes | No | No | IgG | No |
| LMP2A | 1. | LTWRIEDPPFNSLLFA | 0.73 | Yes | No | No | IgG | No |
| BZLF1 | 1. | DSELEIKRYKNRVASR | 0.65 | Yes | No | No | IgG | No |
|  | 2. | MDPNSTSEDVKFTPDP | 0.58 | Yes | No | No | IgG | No |

Ig, Immunoglobulin; In bold, epitopes selected for the multi-epitope vaccine construct.

**Table S11**. Population coverage of five multi-epitope vaccine construct models

| **No** | **Area Geografis** | **Model 1** | **Model 2** | **Model 3** | **Model 4** | **Model 5** |
| --- | --- | --- | --- | --- | --- | --- |
| 1. | Asia Timur | 66,99% | 80,41% | 96,91% | 97,43% | 98,47% |
| 2. | [Asia](http://tools.iedb.org/population/result/#Northeast Asia) Timur Laut | 59,87% | 83,7% | 93,6% | 93,92% | 94,74% |
| 3. | [Asia](http://tools.iedb.org/population/result/#South Asia) Selatan | 71,21% | 85,26% | 95,27% | 95,79% | 96,78% |
| 4. | [Asia](http://tools.iedb.org/population/result/#Southeast Asia) Tenggara | 57,24% | 77,31% | 92,81% | 93,05% | 94,11% |
| 5. | Eropa | 84,55% | 91,74% | 98,08% | 98,72% | 92,87% |
| 6. | Afrika Timur | 74,46% | 77,68% | 92,79% | 93,79% | 99,22% |
| 7. | Afrika Barat | 71,41% | 77,61% | 93,73% | 95,7% | 94,03% |
| 8. | Afrika Tengah | 64,87% | 71,4% | 89,12% | 90,93% | 96,41% |
| 9. | Afrika Utara | 75,12% | 90,29% | 95,73% | 96,67% | 91,46% |
| 10. | Afrika Selatan | 50,78% | 75,68% | 86,36% | 89,03% | 97,05% |
| 11. | Amerika Utara | 75,12% | 90,29% | 98,41% | 98,83% | 99,3% |
| 12. | Amerika Selatan | 55,76% | 75,68% | 90,48% | 91,19% | 91,82% |
| 13. | [Oceania](http://tools.iedb.org/population/result/#Oceania) | 50,91% | 72,4% | 93,66% | 94,33% | 94,83% |
| 14. | [Australia](http://tools.iedb.org/population/result/#Australia) | 48,85% | 80,41% | 89,06% | 90,59% | 91,06% |
| 15. | [Indonesia](http://tools.iedb.org/population/result/#Indonesia) | 50,44% | 83,9% | 89,11% | 89,3% | 89,79% |
| 16. | [Papua New Guinea](http://tools.iedb.org/population/result/#Papua New Guinea) | 49,19% | 67,78% | 98,66% | 98,66% | 98,85% |

**Table S12.** Properties of the multiepitope vaccine construction with the OV-ASP-1 adjuvant

| **Multi-epitope Vaccine**  **Properties** | | **Design Multi-eitope Vaccine** | | | | |
| --- | --- | --- | --- | --- | --- | --- |
|  |  | O1 | O2 | O3 | O4 | O5 |
| Length | | 410 | 574 | 676 | 778 | 880 |
| Antigenicity | | 0.5437  (Antigenic) | 0.6057  (Antigenic) | 0.6175  (Antigenic) | 0.6238  (Antigenic) | 0.6292  (Antigenic) |
| Allergenicity | | Allergen | Allergen | Allergen | Allergen | Allergen |
| Toxicity | | Non-toxic | Non-toxic | Non-toxic | Non-toxic | Non-toxic |
| Physical and  chemical  properties | Molecular weight | 45061.82 | 62305.08 | 73173.07 | 83566.85 | 93984.68 |
|  | Instability  index | 27.77  (Stable) | 27.42  (Stable) | 32.20  (Stable) | 30.52  (Stable) | 31.06  (Stable) |
|  | Aliphatic  index | 66.85  (thermostable) | 64.09  (thermostable) | 61.94  (thermostable) | 63.98  (thermostable) | 64.00  (thermostable) |
|  | GRAVY | -0.616 | -0.612 | -0.650 | -0.580 | -0.539 |
| Solubility | | Soluble | Soluble | Soluble | Soluble | Soluble |
| Population Coverage | World | 78.79% | 94.39% | 97.11 % | 97.74% | 98.46% |
|  | Indonesia | 50.44% | 83.9% | 89.11% | 89.3% | 89.79% |

**Table S13.** Properties of the multiepitope vaccine construction with the Human beta-defensin-3 adjuvant

| **Multi-epitope Vaccine**  **Properties** | | Design Multi-eitope Vaccine | | | | |
| --- | --- | --- | --- | --- | --- | --- |
|  |  | B1 | B2 | B3 | B4 | B5 |
| Length | | 235 | 399 | 501 | 603 | 705 |
| Antigenicity | | 0.6709  (Antigenic) | 0.7077  (Antigenic) | 0.7029  (Antigenic) | 0.6965  (Antigenic) | 0.6928  (Antigenic) |
| Allergenicity | | Allergen | Non-Allergen | Non-Allergen | Non-Allergen | Non-Allergen |
| Toxicity | | Non-toxic | Non-toxic | Non-toxic | Non-toxic | Non-toxic |
| Physical  and  chemical  properties | Molecular  weight | 25712.66 | 42955.92 | 53823.92 | 64217.69 | 74635.52 |
|  | Instability  index | 40.59  (unstable) | 34.82  (stable) | 39.76  (stable) | 36.32  (stable) | 36.15  (stable) |
|  | Aliphatic  index | 63.15  (Non-  thermostable) | 60.70  (Non-  thermostable) | 58.48  (Non-  thermostable) | 61.71  (Non-  thermostable) | 62.06  (Non-  thermostable) |
|  | GRAVY | -0.796 | -0.716 | -0.746 | -0.640 | -0.579 |
| Solubility | | Soluble | Soluble | Soluble | Soluble | Soluble |
| Population Coverage | World | 78.79% | 94.39% | 97.11 % | 97.74% | 98.46% |
|  | Indonesia | 50.44% | 83.9% | 89.11% | 89.3% | 89.79% |

**Table S14.** Properties of the multiepitope vaccine construction without adjuvant

| **Multi-epitope Vaccine**  **Properties** | | Design Multi-eitope Vaccine | | | | |
| --- | --- | --- | --- | --- | --- | --- |
|  |  | A1 | A2 | A3 | A4 | A5 |
| Length | | 190 | 349 | 501 | 553 | 655 |
| Antigenicity | | 0.7083  (antigenic) | 0.7327  (antigenic) | 0.7216  (antigenic) | 0.7112  (antigenic) | 0.7049  (antigenic) |
| Allergenicity | | Non-Allergen | Non-Allergen | Non-Allergen | Non-Allergen | Non-Allergen |
| Toxicity | | Non-toxic | Non-toxic | Non-toxic | Non-toxic | Non-toxic |
| Physical  and  chemical  properties | Moleculer  weight | 20098.95 | 37342.22 | 48210.21 | 58603.98 | 69021.81 |
|  | Instability  index | 8.31  (Stable) | 32.78  (Stable) | 38.73  (Stable) | 35.17  (Stable) | 35.17  (Stable) |
|  | Aliphatic index | 62.27  (Non-  thermostable) | 59.89  (Non-  thermostable) | 57.61  (Non-  thermostable) | 61.28  (Non-  thermostable) | 61.73  (Non-  thermostable) |
|  | GRAVY | -0.830 | -0.722 | -0.755 | -0.640 | -0.572 |
| Solubility | | Soluble | Soluble | Soluble | Soluble | Soluble |
| Population  Coverage | World | 78.79% | 94.39% | 97.11 % | 97.74% | 98.46% |
|  | Indonesia | 50.44% | 83.9% | 89.11% | 89.3% | 89.79% |

**Table S15**. GalaxyRefine Prediction Results (R2,R3,R4,R5)

| **R2** | | | |  |  |  |  |
| --- | --- | --- | --- | --- | --- | --- | --- |
| **Model** | **GDT-HA** | **RMSD** | **MolProbity** | **Clash score** | **Poor rotamers** | **Rama**  **favored** |  |
| Initial | 1.0000 | 0.000 | 0.687 | 0.6 | 0.0 | 98.5 |  |
| MODEL 1 | 0.9525 | 0.411 | 1.025 | 2.4 | 0.0 | 99.0 |  |
| **MODEL 2** | **0.9592** | **0.384** | **0.990** | **2.2** | **0.8** | **98.8** |  |
| MODEL 3 | 0.9458 | 0.417 | 1.087 | 3.0 | 0.5 | 98.5 |  |
| MODEL 4 | 0.9566 | 0.394 | 1.101 | 3.1 | 0.0 | 98.5 |  |
| MODEL 5 | 0.9571 | 0.391 | 1.101 | 3.1 | 0.3 | 98.5 |  |
| **R3** | | | | | | |  |
| **Model** | **GDT-HA** | **RMSD** | **MolProbity** | **Clash score** | **Poor rotamers** | **Rama**  **favored** |  |
| Initial | 1.0000 | 0.000 | 0.721 | 0.7 | 0.0 | 98.1 |  |
| MODEL 1 | 0.9637 | 0.367 | 1.186 | 4.0 | 0.2 | 98.8 |  |
| MODEL 2 | 0.9684 | 0.364 | 1.177 | 3.9 | 0.0 | 98.8 |  |
| **MODEL 3** | **0.9697** | **0.358** | **1.103** | **3.1** | **0.2** | **99.0** |  |
| MODEL 4 | 0.9578 | 0.388 | 1.177 | 3.9 | 0.2 | 99.0 |  |
| MODEL 5 | 0.9637 | 0.384 | 1.167 | 3.8 | 0.2 | 99.0 |  |
| **R4** |  |  |  |  |  |  |  |
| **Model** | **GDT-HA** | **RMSD** | **MolProbity** | **Clash score** | **Poor rotamers** | **Rama**  **favored** |  |
| Initial | 1.0000 | 0.000 | 0.959 | 1.7 | 0.2 | 97.8 |  |
| **MODEL 1** | **0.9517** | **0.400** | **1.055** | **2.7** | **0.0** | **98.1** |  |
| MODEL 2 | 0.9469 | 0.427 | 1.065 | 2.8 | 0.4 | 98.5 |  |
| MODEL 3 | 0.9484 | 0.415 | 1.076 | 2.9 | 0.0 | 98.4 |  |
| MODEL 4 | 0.9422 | 0.436 | 1.086 | 3.0 | 0.2 | 98.7 |  |
| MODEL 5 | 0.9506 | 0.410 | 1.097 | 3.1 | 0.4 | 98.3 |  |
| **R5** |  |  |  |  |  |  |  |
| **Model** | **GDT-HA** | **RMSD** | **MolProbity** | **Clash score** | **Poor rotamers** | **Rama**  **favored** |  |
| Initial | 1.0000 | 0.000 | 0.675 | 0.5 | 0.2 | 98.7 |  |
| MODEL 1 | 0.9570 | 0.405 | 0.892 | 1.5 | 0.3 | 99.1 |  |
| **MODEL 2** | **0.9589** | **0.391** | **1.013** | **2.3** | **0.2** | **99.1** |  |
| MODEL 3 | 0.9516 | 0.411 | 0.944 | 1.8 | 0.5 | 99.4 |  |
| MODEL 4 | 0.9509 | 0.412 | 0.993 | 2.2 | 0.5 | 99.4 |  |
| MODEL 5 | 0.9494 | 0.412 | 0.980 | 2.1 | 0.0 | 99.2 |  |

**Table S16**. Prochechk Prediction Results (R2,R3,R4,R5)

|   R2 |   R3 |
| --- | --- |
|   R4 |   R5 |

**Table S17**. ClusPro Prediction Results

| **Cluspro** | | **Construct Vaccine Multi-epitope** | | | |
| --- | --- | --- | --- | --- | --- |
|  |  | R2 | R3 | R4 | R5 |
| Reseptor | TLR2 | -1131,4  (Model 4) | -1340  (Model 6) | -1408,1  (Model 1 | -1481  (Model 5) |
|  | TLR4 | -1334,2  (model 6) | -1587,7  (Model 15) | -1500,8  (Model 3) | -1629  ( Model 8) |

**Table S18**. HDOCK Prediction Results

| **Construct vaccine** | **Reseptor** | **Model** | **Binding Energy** |
| --- | --- | --- | --- |
| R2 | TLR2 | 1 | 0,9702 |
|  | TLR4 | 1 | 0,9552 |
| R3 | TLR2 | 1 | 0,9893 |
|  | TLR4 | 1 | 0,9783 |
| R4 | TLR2 | 1 | 0,9963 |
|  | TLR4 | 1 | 0,9565 |
| R5 | TLR2 | 1 | 0,9819 |
|  | TLR4 | 1 | 0,9931 |

**Table S18**. Analyzing discontinuous B-cell epitopes

| **No.** | **Residues** | **Number of residues** | **Score** |
| --- | --- | --- | --- |
| 1 | A:K696, A:G697, A:G698, A:R699, A:P700, A:G701, A:A702, A:P703, A:G704, A:G705, A:S706, A:G707, A:S708, A:G709, A:P710, A:R711 | 16 | 0.984 |
| 2 | A:R686, A:S687, A:P688, A:S689, A:S690, A:Q691, A:S692, A:S693, A:S694, A:K695 | 10 | 0.954 |
| 3 | A:K715, A:K716, A:G717, A:G718, A:W719, A:F720, A:G721, A:K722, A:H723, A:R724, A:G725, A:Q726, A:G727, A:G728, A:S729, A:N730 | 16 | 0.947 |
| 4 | A:T781, A:P782, A:D783, A:P784, A:H785, A:H786, A:H787, A:H788, A:H789, A:H790 | 10 | 0.908 |
| 5 | A:K731, A:K732, A:L733, A:T734, A:W735 | 5 | 0.86 |
| 6 | A:H672, A:D674, A:G675, A:V676, A:K677, A:K678, A:G679, A:R680, A:G681, A:E682, A:K683, A:R736, A:I737, A:E738, A:D739, A:P740, A:P741, A:F742, A:N743, A:S744, A:L745, A:L746, A:F747, A:A748, A:K749, A:K750, A:D751, A:S752, A:E753, A:L754, A:E755, A:I756, A:K757, A:R758, A:Y759, A:K760, A:N761, A:R762, A:V763, A:A764, A:S765, A:R766, A:K767, A:K768, A:M769, A:D770, A:P771, A:N772, A:S773, A:T774, A:S775, A:E776, A:D777, A:V778, A:K779, A:F780 | 56 | 0.825 |
| 7 | A:V34, A:T35, A:A36, A:A37, A:A38, A:P39, A:V40, A:A41, A:V42, A:A43, A:A44, A:A45, A:G46, A:A47, A:A48, A:P49, A:A50, A:G51, A:A52, A:A53, A:V54, A:E55, A:A56, A:A57, A:E58, A:E59, A:Q60, A:S61, A:E62, A:F63, A:D64, A:V65, A:I66, A:L67, A:E68, A:A69, A:A70, A:G71, A:D72, A:K73, A:K74, A:I75, A:G76, A:V77, A:I78, A:V81 | 46 | 0.745 |
| 8 | A:A176, A:V177, A:L178, A:T179, A:L180, A:A181, A:A182, A:Y183, A:M184, A:L185, A:V186, A:L187, A:Q188, A:W189, A:A190, A:S191, A:L192, A:A193, A:A194, A:Y195, A:R196, A:K197, A:K198, A:G199, A:G200, A:W201, A:F202, A:G203, A:K204, A:A205, A:A206, A:Y207, A:R208, A:T209, A:T210, A:D211, A:E212, A:G213, A:T214, A:W215, A:V216, A:A217, A:A218, A:Y219, A:G220, A:L221, A:Q222, A:G223, A:I224, A:Y225, A:V226, A:L227, A:V228, A:A229, A:A230, A:Y231, A:T232, A:F233, A:F234, A:A235, A:I236, A:C237, A:L238, A:T239, A:A241, A:A242, A:G405, A:G407, A:G408, A:W409, A:F410, A:G411, A:K412, A:H413, A:G415, A:Q416, A:G417, A:G418, A:S419, A:N420, A:G421, A:P422, A:G423, A:P424, A:G425, A:K426, A:T427, A:Y430, A:N431, A:R434 | 90 | 0.712 |
| 9 | A:A285, A:S286, A:A287, A:K288, A:A289, A:A290, A:Y291, A:S292, A:E293, A:L294, A:E295, A:I296, A:K297, A:R298, A:Y299, A:K300, A:G301, A:P302, A:G303, A:P304, A:G305, A:T306, A:A307, A:V308, A:V309, A:R310, A:A311, A:Q312, A:G313, A:L314, A:D315, A:V316, A:T317, A:L318, A:P319, A:L320, A:G321, A:P322, A:G323, A:P324, A:G325, A:Q326, A:D327, A:S328, A:N329 | 45 | 0.704 |
| 10 | A:G661, A:A662, A:P663, A:G664, A:G665, A:S666, A:G667, A:S668, A:G669, A:P670 | 10 | 0.698 |
| 11 | A:F330, A:S331, A:V332, A:K333, A:T334, A:E335, A:M336, A:L337, A:G338, A:N339, A:E340, A:G341, A:P342, A:G343, A:P344, A:G345, A:D346, A:S347, A:N348, A:F349, A:S350, A:V351, A:K352, A:T353, A:E354 | 25 | 0.679 |
| 12 | A:K26, A:K27, A:F28, A:E29, A:E30, A:T31, A:F32 | 7 | 0.618 |
| 13 | A:I550, A:K551, A:R552, A:Y553, A:K554, A:N555, A:R556, A:V557, A:A558, A:S559, A:R560, A:G561, A:P562, A:G563, A:P564, A:G565, A:D566, A:S567, A:E570 | 19 | 0.609 |
| 14 | A:M1, A:A2, A:K3, A:L4, A:S5 | 5 | 0.588 |
| 15 | A:R653, A:S654, A:P655, A:S656, A:S657, A:Q658, A:K659, A:K660 | 8 | 0.572 |
| 16 | A:A635, A:K638, A:S639, A:K642, A:R643 | 5 | 0.55 |
| 17 | A:S592, A:A593, A:K594, A:S595, A:S596, A:E597, A:D599 | 7 | 0.52 |
| 18 | A:G648, A:E649, A:K650 | 3 | 0.503 |

**Figure S1** Visualization PyMOL for tertiary structure

The model displays the spatial organization of the vaccine components, including adjuvant (red), HTL epitopes (blue), B-cell epitopes (purple), CTL epitopes (yellow), linker sequences (white), and the 6×His-tag (green).

**Figure S2.** Flexibility analysis of the designed multi-epitope vaccine construct using CABS-flex 2.0.

The predicted structural fluctuations and residue-level flexibility of the vaccine model are shown as root mean square fluctuation (RMSF) values, indicating regions with higher conformational mobility.

**Figure S3**. Docking interaction of the multi-epitope vaccine construct with TLR2 and TLR4 receptors.
The predicted binding affinity between the designed vaccine construct and TLR2/TLR4 was evaluated using the HDOCK server. Docking scores were categorized as high (>0.7), intermediate (0.5–0.7), and low (<0.5) predicted binding affinity. The results illustrate the comparative interaction profiles of the vaccine construct with both receptors.

receptors.

**Figure S4.** Prediction of R5 Multi-epitope Vaccine Normal mode analysis (NMA) using the Imods server.

Molecular dynamics prediction was performed using iMODS to evaluate the deformability and structural stability of the vaccine–receptor complexes. The calculated eigenvalues were 2.68 × 10⁻⁷ for the vaccine TLR2 complex and 7.69 × 10⁻⁷ for the vaccine TLR4 complex, suggesting differences in intrinsic flexibility between the two complexes.
